# Immunoregulatory electrospinning fiber mediates Macrophage energy metabolism reprogramming to promote burn wound healing

**DOI:** 10.1016/j.mtbio.2025.102430

**Published:** 2025-10-16

**Authors:** Haoyang Wu, Qimeng Wu, Chen Liang, Jiali Hua, Lingyi Meng, Paweł Nakielski, Chenyan Lu, Filippo Pierini, Liqun Xu, Yunlong Yu, Qianqian Luo

**Affiliations:** a Department of Hypoxic Biomedicine, Institute of Special Environmental Medicine and Coinnovation Center of Neuroregeneration, Nantong University, 226019, Nantong, PR China; b Institute of Burn Research, Southwest Hospital, Third Military Medical University (Army Medical University), 400038, Chongqing, PR China; c BRICS Joint Laboratory on Biomedical Materials, School of Materials and Energy, Southwest University, 400715, Chongqing, PR China; d Multidisciplinary Centre for Advanced Materials, Institute for Frontier Medical Technology, School of Chemistry and Chemical Engineering, Shanghai University of Engineering Science, 201620, Shanghai, PR China; e Department of Biosystems and Soft Matter, Institute of Fundamental Technological Research, Polish Academy of Sciences, 02-106, Warsaw, Poland

**Keywords:** Burn, Macrophage, Wound healing, Metabolism reprogramming, OXPHOS

## Abstract

Burn wound management posed substantial therapeutic challenges due to impaired macrophage polarization dynamics. Metabolic dysfunction in macrophages hindered the transition from glycolysis-driven M1 phenotype to oxidative phosphorylation (OXPHOS)-driven M2 phenotype, result of perpetuating inflammatory reaction to restrain wound healing. Despite all kinds of biomaterials were developed for burn wounds, some critical issues still couldnot be solved, such as limited repair efficacy, strong immunogenicity, and high cost *etc*. Cellular metabolite α-ketoglutaric acid (AKG) shows good biological activity and can regulate cellular energy metabolism, which is expected to solve the above issues. However, the cellular acid-toxicity of AKG might restrict its wide application in clinic. Therefore, a bioactive electrospinning fiber (PEKUU) was engineered to demonstrate sustained AKG release for modulation of energy metabolism of burn wounds. *In vitro* assessments confirmed its biocompatibility and effects on keratinocyte and endothelial proliferation, migration and angiogenesis. Meanwhile, PEKUU could attenuated glycolysis-driven M1 polarization, reducing NF-κB-mediated inflammation. While it also could enhance mitochondrial OXPHOS to drive M2 polarization. *In vivo* experiment showed that PEKUU electrospinning fiber could accelerate epithelialization, collagen remodeling and healing of deep second-degree burn wounds of mice. Finally, proteomics was applied to reveal the underlying mechanism of AKG-mediated metabolic reprogramming, including the coordinated suppression of the glycolytic-NF-κB axes and the potentiation of the OXPHOS and fatty acid oxidation pathways. The dual regulation reshaped macrophage energetics and established a pro-regenerative niche. Overall, PEKUU electrospinning dressing could modulate macrophage polarization state by reprogramming energy metabolism mode, providing a new therapeutic strategy for burn repair.

## Highlights

• In order to reduce the acid toxicity of AKG, bulk polymerization reaction was conducted to load AKG in polyurethane for controlled release.
• Bioactive metabolite AKG showed efficient energy metabolism programming to regulate macrophage polarization for burn wound healing.
• Proteomics analysis proved that AKG could promote OXPHOS and FAO through PPAR-γ and STAT3 to modulate M2-phenotype translation.

## Graphical abstract

**Figure:**
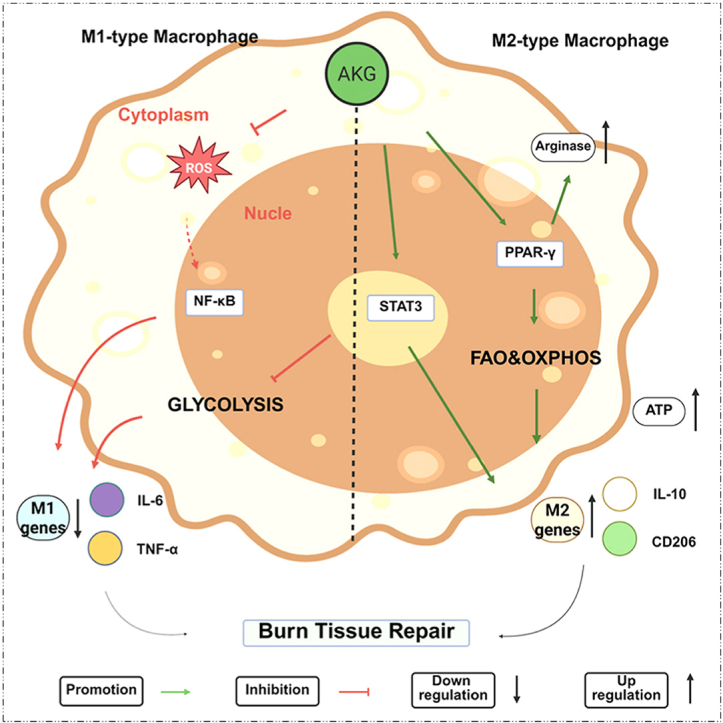

## 1 Introduction

**Scheme 1:**
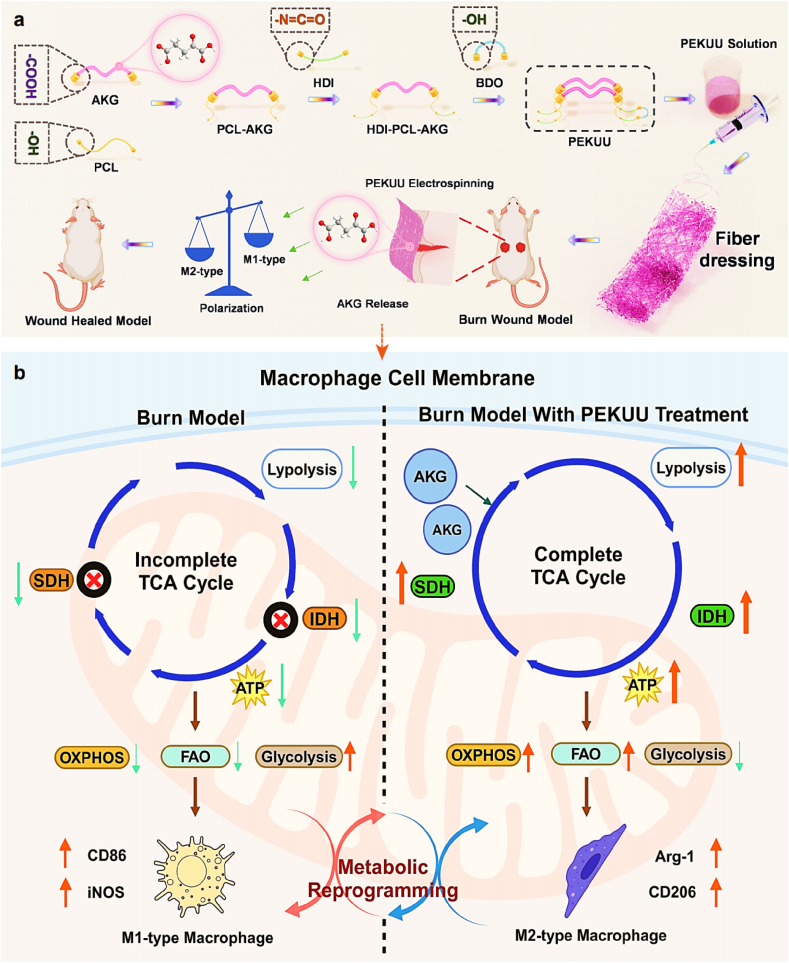
Schematic diagram of burn wound healing process treated by PEKUU electrospinning fiber. (a) Schematic diagram of the reaction sequence for the synthesis of PEKUU. (b) Macrophage metabolic reprogramming effect after PEKUU treatment on burn wound.

Burn injury has become a critical global public health concern due to the profound pathophysiological impact. The direct destruction of deep dermal structures results in extensive cellular damage and functional impairment at wound sites [1], compounded by dysregulated inflammatory homeostasis [2,3]. Those pathomechanisms render burn wound healing more complex than conventional wound, imposing severe quality-of-life burdens on survivors. In clinic, severe burns frequently result in systemic complications, including hypovolemic shock and sepsis, with mortality rates exceeding 20 % [4]. Even wounds that are successfully healed often associated with hypertrophic scarring and functional deficits. According to the epidemiological data published by the World Health Organization (WHO) [5], burn patients suffer from approximately 180,000 annual deaths on a global scale. Furthermore, the incidence of mortality and disability rate associated with burn injury consistently rank among the top 15 causes of global disease burden [[5], [6], [7]]. Thus, it is urgent to develop effective therapeutic strategies to improve the healing rate and quality of burn wounds (see Scheme 1).

The progression of burn repair is characterized by a dynamic cascade of coordinated biological events, requiring precise spatiotemporal regulation of inflammatory responses, proliferative phases, and tissue remodeling processes [8,9]. Macrophages emerged as indispensable immunoregulators in wound repair, orchestrating multiple biochemical and cellular reactions during healing [10]. The polarization phenotypes of macrophages were found to be directly governed by metabolic models. Specifically, glycolytic flux predominantly drove pro-inflammatory M1 polarization [11], while fatty acid oxidation (FAO) and oxidative phosphorylation (OXPHOS) were essential for anti-inflammatory M2 polarization and tissue regeneration [12]. Cellular energy has been identified as a critical factor for wound healing [13,14]. The inflammatory microenvironment present in burn wound had been shown to preferentially activate pro-inflammatory M1-phenotype macrophages, prompting a shift in their metabolic adaptation from OXPHOS to glycolysis [[15], [16], [17]], which was known to be inefficient in adenosine triphosphate (ATP) generation [18,19]. Thus, decrease of ATP supplement caused delayed burn wound healing [20,21]. Previous research found that there were two breakpoints in tricarboxylic acid cycle (TCA) of M1, which were isocitrate dehydrogenase (IDH) and succinate dehydrogenase (SDH) [22]. The enzymatic deficiencies of IDH and SDH restricted M2 polarization, further resulting in impaired healing of burn wound. Thereby, it would be a promising strategy to regulate macrophage phenotype polarization through mediating energy metabolism reprogramming.

In recent decades, lots of therapeutic strategies for burn wound healing had been extensively investigated, including stem cell-based therapies [23], pharmacological interventions and biomaterial treatments, *etc* [24]. Especially, the advent of biomaterial engineering had achieved significant advancements, such as polypeptide for angiogenesis promotion [25,26], advanced fibrous dressings [27], and antimicrobial nanoparticles [28,29]. However, these approaches were frequently constrained by inherent limitations, including toxicity, immunogenicity and cost. To solve those issues, endogenic cellular metabolites might be chosen as the therapeutic agents [30,31]. α-Ketoglutarate (AKG), a central TCA cycle intermediate [31], has been demonstrated multifaceted therapeutic potential through its involvement in collagen biosynthesis [32], oxidative stress mitigation [33], anti-inflammatory regulation [34], cellular proliferation enhancement [35], and epigenetic modulation [36]. The above involved processes are critical to wound healing cascades, which endows AKG a potential metabolic regulator for tissue regeneration [14,37]. However, the direct topical application of AKG is compromised by pH-mediated toxicity stemming from its acidic properties. Consequently, development of sustained-release carrier system emerges as a critical solution to achieve localized AKG delivery and metabolic regulation.

Fibrous dressings were frequently employed in clinic due to their inherent high surface area-to-volume ratio and porous architectures [38,39], which effectively absorbed wound exudates to maintain a moist microenvironment and substrates for cellular adhesion and proliferation. With the knowledge learned above, a multifunctional electrospinning fibrous dressing (PEKUU) was engineered to enable the sustained release of AKG for regulation of macrophage metabolic reprogramming. A comprehensive material characterization was performed to test the physicochemical properties of the prepared fibers using analytical techniques. *In vitro* biocompatibility assessments of PEKUU demonstrated good cell viability, with enhanced keratinocyte proliferation, fibroblast migration, and angiogenesis. Molecular biological techniques and Seahorse were applied to reveal that PEKUU could effectively augment mitochondrial OXPHOS activity in macrophages while suppressing glycolytic flux. Thereby M1 was polarized towards M2 to produce more ATP for cells. *In vivo* evaluation confirmed that PEKUU could alleviate inflammatory factors, enhance lipid mobilization, shift macrophages from M1 to M2 types, and induce orderly collagen deposition. Ultimately, accelerated wound healing was dramatically achieved in rat burn models. Furthermore, proteomics were conducted to reveal the potential metabolic regulatory mechanism of burn repair after PEKUU treatment. The above findings established a novel metabolism-directed strategy for burn management, presenting significant translational potential across clinical scenarios requiring immunometabolic intervention.

## 2 Materials and methods

### 2.1 Synthesis of PU

The synthesis of PU was conducted via a two-step polymerization method. Initially, PCL-diol was reacted with hexamethylene HDI at 80 °C under a nitrogen atmosphere for 3 h to form a prepolymer. Subsequently, BDO was added as a chain extender, and the chain extension reaction proceeded for 18 h. The molar ratio of PCL-diol, HDI, and BDO was maintained at 1:2:1. Upon completion of the reaction, the product was purified by repeated washing with deionized water for 3 days, followed by freeze-drying to obtain the final PU material.

### 2.2 Synthesis of PEKUU

PEKUU was synthesized using PCL-diol, AKG, HDI, and BDO at a molar ratio of 2:1:2:1. Initially, PCL-diol and AKG were subjected to cyclic dehydration under vacuum at 140 °C for five cycles to eliminate residual moisture. Subsequently, HDI and an organotin catalyst were sequentially introduced into the reaction system under a nitrogen atmosphere, followed by a prepolymerization step at 80 °C for 3 h to form the prepolymer. During the chain-extension stage, BDO dissolved in dimethyl sulfoxide (DMSO) was slowly added via dropwise infusion, and the reaction proceeded for 8 h. The viscosity of the system was monitored in real time to dynamically adjust the DMSO supplementation, ensuring consistent solution fluidity throughout the process. The final product was purified by precipitation in deionized water for 3 days and freeze-dried to yield PEKUU.

### 2.3 Preparation of the electrospinning solution

Dissolve the appropriate amount of PEKUU in Hexafluoroisopropanol (HFIP) so that the final concentration of the electrospinning solution is 20 % by mass volume. Dissolve the appropriate amount of PU in HFIP and ensure the final concentration of 20 %, after thorough stirring, separate the bottles, accurately weigh α-ketoglutaric acid to add one bottle, the final preparation of α ketoglutaric acid and PU mass ratio of 5.88 % of PU/AKG electrospinning solution, and continue stirring until the α-ketoglutaric acid is completely dissolved.

### 2.4 Preparation of electrospinning

HFIP was utilized as the solvent to prepare homogeneous electrospinning solutions of PU and PEKUU at a concentration of 20 % (w/v). For the PU/AKG blended electrospinning solution, 5.88 % (w/w) AKG was uniformly incorporated into the PU solution under magnetic stirring until complete dissolution. Electrospinning was carried out under a high-voltage electric field of 10 kV, with the solutions extruded through a 20-gauge needle at a controlled flow rate of 1.0 mL/h. A fixed collection distance of 15 cm was maintained to fabricate three distinct nanofibrous membranes: PU, PU/AKG, and PEKUU.

### 2.5 Characterisation

Microstructural characterisation: The surface morphology of the fibres was characterised by field emission scanning electron microscopy (FESEM, JSM-7800F, JEOL Ltd., Tokyo, Japan). The diameter of the fibres was calculated using Image J. One hundred fibres were selected for each sample to obtain a statistical distribution of diameters and the mean values were used for data analysis.

FTIR: The chemical structure and composition of the fibre dressings were characterised in detail by FTIR testing. FTIR of fibres is measured in the wave number range of 4000 to 600 cm (Spectrum Two™, PerkinElmer).

X-ray photoelectron spectroscopy: by XPS analysis (K-alpha, Thermo Fisher Scientific) to determine the surface elemental composition of fibres such as PU.

### 2.6 Dissolution

Fibres (initial weight 50 mg, expressed as W0) were dispersed in 100 mL PBS at 37 °C for 12 h. After hygroscopic swelling, the fibres were removed and weighed and the weight was expressed as Ws. The swelling rate formula is given below:

$$Swelling\;rate=\frac{Ws-W0}{W0}*100\%$$

Each sample was tested at least 5 times and the data were analysed using the mean values.

### 2.7 *In vitro* drug release

The drug release properties were tested *in vitro*. The drug-carrying fibre (50 mg) was dispersed in 5 mL of PBS, pH 7.4, as a release medium. Approximately 1 mL of sample was removed from the release medium at regular intervals and fresh medium was added to maintain a constant volume. The standard curve of ketoglutarate was measured using a UV–Vis absorption spectrophotometer (Lambda 365, Perkin Elmer) and the concentration of ketoglutarate in the release medium was calculated. The concentration of ketoglutarate was determined by measuring the absorbance at 235 nm. Each sample was analysed at least five times.

### 2.8 *In vitro* cell proliferation and migration assay

For the CCK-8 assay, human umbilical vein endothelial cell line HUVEC cells and human immortalised keratinocyte cell line Hacat cells were inoculated into 24-well plates at a density of 3000 cells/well and then co-cultured overnight by Transwell with electrospinning at 37 °C and 5 % CO2. Cell viability was then assessed using the CCK-8 method according to the manufacturer's instructions (C0038, Beyotime Biotechnology, China). Absorbance was measured at 450 nm using a SpectraMax® M2e microplate reader (Molecular Devices, USA). Cell viability was calculated according to the following formula.

$$\text{Cell}\;\text{viability}\;\left(\%\right)=\left[\left({\text{OD}}_{\text{Sample}}-{\text{Od}}_{\text{Blank}}\right)/\left({\text{OD}}_{\text{Ctrl}}-{\text{OD}}_{\text{Blank}}\right)\right]x\;100\%\text{.}$$

Where OD_Sample_ represents the OD value of the experimental material group; OD_Blank_ represents the OD value of the culture medium; OD_Ctrl_ represents the OD value of the control group.

In addition, the live/dead staining test using a kit (Proteintech, PF00007, China) was used to assess cell viability on day 3. Under a fluorescence microscope (Olympus, Japan), live cells stained with calcium fudge showed a green signal, while dead cells stained with propidium iodide (PI) showed a red signal.

For cell migration assays, 3 × 10^4^ L929 cells/well were inoculated into 24-well plates and grown to confluence. First, the bottom of each well was carefully scraped and 6 free cells were gently washed with PBS. Pure medium without FBS was then added to each well and 1.6 μM PU, PU/AKG and PEKUU were electrostatically spun in a Transwell insert to co-incubate with the cells. Cells treated only with a blank Transwell were used as controls. Cells were imaged using a fluorescence microscope (Olympus, Tokyo, Japan) at 0 and 24 h and the scratch width was measured using ImageJ software. The migration rate (%) was calculated as follows

$$\text{Migration}\;\text{rate}\;\left(\%\right)=\left[\left(L_{0}-L_{24}\right)/L0\right]\times 100\%\text{.}$$

where L_0_ represents the initial width of the scratch and L_24_ represents the final width of the scratch after 24 h of co-incubation.

### 2.9 Hemolysis experiment

Anticoagulated rat blood was centrifuged at 1000 rpm for 10 min to obtain red blood cells (RBCs). The RBCs were then washed three times with PBS and diluted with PBS to a final concentration of 5 % (v/v). Then 500 μl of diluted RBCs were incubated with 1.6 μM PU, PU/AKG and PEKUUA electrospinning. RBCs incubated with deionized water and PBS were used as positive and negative controls, respectively.

After incubation at 37 °C for 1 h, RBCs from each group were centrifuged at 1000 rpm for 5 min. The supernatants were then aspirated and the absorbance of each group was measured at 545 nm using a microplate reader. The hemolysis ratio was calculated according to the following formula:

$$\text{Hemolysis}\;\text{ratio}\;\left(\%\right)=\left[\left(A_{\text{Sample}}-A_{\text{PBS}}\right)/\left(A_{\text{Water}}-A_{\text{PBS}}\right)\right]x\;100\%\text{.}$$

Where A_Sample_ represents the absorbance of PU, PU/AKG, PEKUU groups; A_PBS_ represents the absorbance of phosphate buffer group; A_Water_ represents the absorbance of deionized water group.

### 2.10 Tubule formation assay

HUVECs were inoculated into 24-well plates with Matrigel at 1.5 × 10^5^ per well in serum-free medium. The plates were incubated at 37 °C for 24 h. The cells were then photographed under a microscope and photo-analysed, and the total tubule length was measured using ImageJ.

### 2.11 Isolation of BMDMs

Femurs and tibiae of C57BL/6 mice were harvested for bone marrow cell isolation. Extracted bone marrow cells were rinsed with phosphate buffered saline (PBS) and excess tissue debris was filtered through a 70 μm cell filter to obtain a pure single cell suspension. Erythrocytes were removed using ACK lysis buffer. The cells were then cultured in the presence of macrophage colony stimulating factor (20 ng/ml, ABclonal, China) for 5 days to induce differentiation into BMDMs.

### 2.12 *In vitro* antioxidant assays

Intracellular ROS scavenging properties were assessed using a ROS probe according to the manufacturer's instructions (S0035S, Beyotime Biotechnology, China). Bone marrow-derived macrophages (BMDMs) were isolated from C57BL/6 mice and cultured in RPMI 1640 medium containing 10 % FBS and 20 ng/ml M-CSF. BMDM were concentrated at 5 × 10^5^ cells/well for 6-well plates and then treated with fresh medium containing H_2_O_2_ (0.5 mM) and co-incubated with electrostatic spins placed in Transwell at 37 °C. Cells treated with fresh medium were used as negative controls, while cells treated with H_2_O_2_-containing medium were used as positive controls. After 6 h of incubation, the culture medium was removed by washing with serum-free medium according to the manufacturer's instructions, washed three times with PBS, and then the cells were stained with a DCFH-DA probe (S0035S, Beyotime Biotechnology, China). Cells were observed and imaged using CLSM (Olympus, Japan), with green fluorescence indicating ROS, and data were analysed using ImageJ software.

### 2.13 Macrophage polarization

BMDMs at 5 × 10^5^ cells/well were seeded on cell crawls in 24-well plates and stimulated with LPS (200 ng/ml) for 24 h to polarization to the M1 phenotype. Subsequently, 1.6 μM PU, PU/AKG and PEKUU electrospinning fiber were set in Transwell and co-incubated with cells for 24 h. LPS-stimulated BMDMs without further treatment were used as Ctrl.

For immunofluorescence staining, cells were fixed in 4 % paraformaldehyde for 30 min and then washed 3 times with PBS. The cells were then incubated overnight at 4 °C with rabbit monoclonal antibodies against Arg-1 (A25808, Abclonal, China) and against iNOS (ab283655, Abcam, UK). After three washes with PBS, FITC-conjugated goat anti-mouse IgG secondary antibody (A0423, Beyotime Biotechnology, China) was incubated with the cells on a shaker at room temperature. The cells were washed with PBS and stained with 4′,6-diamidino 2-phenylindole (DAPI, C1002, Beyotime Biotechnology, China). Finally, the cells were imaged and analysed using CLSM, with green fluorescence representing the target protein signal and blue fluorescence representing the nucleus.

### 2.14 Measurement of intracellular ATP levels

To determine intracellular ATP levels in primary bone marrow macrophages, BMDMs were cultured in six-well culture plates to 85 %–95 % density, then LPS was added to the medium and co-cultured with primary bone marrow macrophages by Transwell electrospinning. After 24 h, the cells were lysed and ATP levels were determined using the ATP Assay Kit according to the manufacturer's instructions (S0026, Beyotime Biotechnology, China), n = 3.

### 2.15 Measurement of intracellular IDH levels

To determine intracellular IDH levels in primary bone marrow macrophages, BMDMs were cultured in six-well culture plates to a density of 85 %–95 %, and then LPS was added to the medium and allowed to co-cultivate with primary bone marrow macrophages by Transwell for electrospinning. The cells were lysed after 24 h and IDH levels were determined using an IDH assay kit according to the manufacturer's instructions (S0526S, Beyotime Biotechnology, China) n = 3.

### 2.16 Measurement of intracellular SDH levels

To determine intracellular SDH levels in primary bone marrow macrophages, BMDMs were cultured in six-well culture plates to a density of 85 %–95 %, then LPS was added to the medium and co-cultured with primary bone marrow macrophages in a Transwell to allow electrospinning, and the cells were lysed after 24 h. Cells were lysed and SDH levels were determined using an SDH assay kit according to the manufacturer's instructions (S0530S, Beyotime Biotechnology, China) n = 3.

### 2.17 RT-qPCR

Total RNA was extracted from BMDMs cells using Trizol reagent (15596026, Invitrogen, USA) according to the manufacturer's instructions, and the RNA concentration was measured using a NanoDrop spectrophotometer (Thermo). Then, 1 μg of RNA was reverse transcribed to cDNA by reverse transcription.

RT-qPCR detection and quantification was performed using SYBR Green and the LineGene 9600 Plus Real-Time PCR System (FQD-96A, Bioer, China). Primers used are listed in Table S1.

### 2.18 Western blot

Skin tissues and BMDM cells were collected and homogenised with RIPA lysis buffer (BL504A, Biosharp, China) and then sonicated with an ultrasonograph (Scientz-48L, Xinzhi, China). Protein concentration was determined using a BCA assay kit (U10007A, Koyobo, China). Skin tissue samples containing 20 μg of proteins were loaded onto SDS-PAGE gels for separation and then transferred to PVDF membranes. The protein-containing PVDF membranes were blocked with 5 % skimmed milk and then the PVDF membranes were blocked with various primary antibodies including anti-CD206 (ab300621, Abcam, UK), anti-CD86 (CY5238, Abways, China), anti-PPARγ (41360, SAB, USA), anti-iNOS (ab178945, Abcam, UK), anti-Arg1 (A25808, Abclonal, China), anti-CPT2 (26555-1-AP, Proteintech, China), anti-Hexokinase2 (ab209847,Abcam, UK), anti-PKM2 (4053, CST, USA), anti-Glut1 (21829-1-AP, Proteintech, China), anti-P65 (8242, CST, USA), anti-p-P65 (3033, CST, USA), anti-STAT3 (9139, CST, USA), anti-p-STAT3 (9145, CST, USA), anti-CD163 (ab182422, Abcam, UK), anti-CD36 (sc21772, Santa Cruz, USA) and anti-PCNA (13110, CST, USA) (incubated at a dilution of 1: 1000 overnight at 4 °C. Then incubate with secondary antibody (anti-rabbit or anti-mouse IgG, 1:10,000 dilution, Jackson, 115-035-003) for 1 h at room temperature. The color was developed with an enhanced chemiluminescent substrate (U10010A, Keyoubo, China) and the signal was detected with a chemiluminescent imaging system (4100, Tanon, China). Scanning and analysis were performed using ImageJ software and GraphPad software. Results were expressed as the ratio of optical density to α-tubulin after normalization.

### 2.19 Energy metabolism analysis

To investigate the glycolytic activity and mitochondrial respiratory of macrophages treated by PU, PU/AKG, and PEKUU electrospinning fibers, extracellular acidification rates (ECARs) and mitochondrial oxygen consumption rates (OCRs) were monitored by a Seahorse XFe96 analyzer (Agilent Technologies, USA).

For OCR analysis, the Seahorse XFe96 analyzer was calibrated and the basal OCR was first detected. Then 1.5 μM oligomycin (OLI), 1.0 μM carbonyl cyanide 4-(trifluoromethoxy), phenylhydrazone (FCCP) and 0.5 μM rotenone/antimycin A (ROT/AA) were sequentially injected during the real-time OCR measurements. The respiratory parameters were calculated as follows: basal respiration=(last rate measurement before first injection)-(Non-mitochondrial respiration rate); maximal respiration=(maximum rate measurement after FCCP injection)-(Non-mitochondrial respiration); spare respiratory capacity=(maximum respiration)-(basal respiration); and ATP production=(last rate measurement before oligomycin injection)-(minimum rate measurement after oligomycin injection).

For ECAR analysis, 10 mM glucose, 1 mM oligomycin and 50 mM 2-DG were sequ entially injected during real-time ECAR test. The glycolytic respiration parameters were cal culated as follows: glycolysis=(maximum rate measurement before oligomycin injection)-(la st rate measurement before glucose injection); glycolysis capacity=(maximum rate measure ment after oligomycin injection)-(last rate measurement before glucose injection); glycolysis reserve=(glycolysis capacity)-(glycolysis).

### 2.20 Establishment of the burn model

The SD rats used in this study were provided by the Animal Experiment Centre of Nantong University. The mice were housed in individually ventilated cage systems at a temperature of 21 ± 2 °C, relative humidity of 55–60 %, and 12-h alternating light and dark. All animal treatment procedures were carried out in accordance with the Chinese Animal Management Measures of the Ministry of Health and were approved by the Nantong University Animal Ethics Committee Research Project Protocol (S20250915-005). The study was conducted in accordance with the ARRIVE guidelines.

Twelve SPF grade SD rats, male, weighing 160–200 g, were provided by the Experimental Animal Centre of the Nantong University. They were randomly divided into 4 groups of 5 rats each. Anaesthesia (200 g/mL) was induced by intraperitoneal injection of 3 % sodium pentobarbital one day before the experiment. After anaesthesia, the rats were shaved on the back with a clipper and evenly coated with Viton hair removal cream for hair removal. On the following day, the rats were first anaesthetised, and then a desktop super-temperature controlled scalding instrument (Jinan Yiyan Science and Technology Development Co., Ltd., YLS-5Q) was used with a parameter setting of 85° for 15 s. The probe of the scalding instrument (probe diameter 10 mm) was placed vertically on the back of the rats and pressed for 15 s. The scalded area was pale, then cut off the burded skin in the night, resulting in a deep Ⅱ degree burn model. A control group and an experimental group were established, and in the experimental group, PU, PU/AKG and PEKUU electrospinning silk were applied to the scalded area and fixed with a bandage. Photographs were taken at 0, 3, 7 and 14 days to observe the healing of the scalded wounds and the healing rate was counted using ImageJ software.

### 2.21 Morphological and wound surface analysis

Morphological changes in the burned skin, such as colour, swelling and eschar formation, were assessed by visual inspection of the wounds. Changes in the wound were recorded by camera on days 0, 3, 7 and 14 after burn treatment. On days 0, 3, 7 and 14, the rats were euthanised according to the approved euthanasia protocol and the dorsal skin of the rats was collected for subsequent experiments.

Wound area (%) = A_n_/A_0_ × 100 %.

A_n_ represents the wound area on days 3, 7 and 14 and A_0_ represents the wound area on day 0.

### 2.22 Hematoxylin and eosin (H&E) staining and histological analysis

Hematoxylin and eosin (H&E) staining was used to assess structural changes in the wound skin. Skin tissues were fixed in 4 % paraformaldehyde for 24 h, embedded in paraffin, and then cut into 10 μm thick sections for subsequent staining procedures. H&E staining was performed using the H&E staining kit (C0105S, Beyotime Biotechnology, China).

### 2.23 Masson staining

Masson's staining was performed using a modified Masson's trichrome staining kit (G1346, Solarbio, China). Sections were deparaffinised in xylene and then rehydrated through a series of graded alcohols (30 %, 50 %, 75 %, 80 %, 95 %, 95 %, 100 % and 100 %) for 2 min per step. The sections were then immersed in equal volumes of absolute ethanol and xylene and a mixture of xylene I and xylene II for 15 min. The sections were then stained with hematoxylin, differentiated in an acidic solution and restained with eosin. The sections were then dehydrated in a series of graded alcohols (30 %, 50 %, 75 %, 80 %, 95 %, 95 %, 100 % and 100 %) for 2 min per step and finally embedded in neutral resin. Images were captured using a Leica fluorescence microscope (DM4000B, Leica, Germany).

### 2.24 *In vivo* antioxidant stress experiment

On day 4 after completion of the burn model establishment by electrospinning treatment, wound tissues (1 cm × 1 cm) were collected from each group and sectioned by cryosectioning. Wound ROS staining was then performed using a DCFH-DA probe (S0035S, Beyotime Biotechnology, China) according to the manufacturer's instructions. Finally, the stained sections were observed by CLSM (Olympus, Tokyo, Japan). Green fluorescence represents ROS and blue fluorescence represents nuclei. Fluorescence intensity was quantified using ImageJ software.

### 2.25 Immunohistochemical staining

Paraffin sections (10 μm) of skin tissue were permeabilized with 0.3 % Triton X-100, then blocked with 10 % donkey serum and incubated overnight with primary antibodies aga inst IL-10 (60269-1-Ig, Proteintech, China), IL-6 (ab11575, Abcam, UK), TNFα (11948, CST, USA), CD31 (ab28364, Abcam, UK). Skin sections were then sequentially incubated with secondary ant ibodies, reaction enhancer and 3,3′-diaminobenzidine (DAB, Fuzhou, China). Tissue images were captured using a fluorescence microscope (DM4000B, Leica, Germany) and analysed using ImagingJ software.

### 2.26 *In vivo* immunofluorescence staining

Paraffin sections were deparaffinised by gradual dehydration in a citrate buffer bath at 100 °C, then hydrated and boiled. The sections were then incubated with primary antibodies including rabbit anti-iNOS antibody (ab283655, Abcam, UK), rabbit anti-F4/80 antibody (28463-1 AP, Proteintech, China) and rabbit anti-Arg-1 antibody (A25808, Abclonal, China). After overnight incubation at 4 °C, wound sections were washed with PBS and incubated with Alexa 488 goat anti-rabbit IgG (Beyotime Biotechnology, China) for 1 h. After co-staining with DAPI dye, sections were imaged and analysed using Confocal. (Olympus, Tokyo, Japan).

### 2.27 Proteomic analysis

Label-free proteomic analyses were performed as previously described. Briefly, control tissues and PEKUU wound tissues were harvested on postoperative day 7 (n = 3) and immediately treated with liquid nitrogen. Total proteins were extracted and protein concentrations were measured using the Bradford method (Thermo Fisher Scientific, USA). Qualified peptide samples were digested with trypsin and desalted using a C18 column (WAT054955, Waters, USA) and then analysed using a NanoLC-ESI-MS/MS system on the Thermo Scientific Orbitrap Exploris™480 platform (Thermo Fisher Scientific, at Sichuan Panomix Biotechnology Co. USA) was used to identify and quantify proteins using the Proteome Discoverer suite (version 2.4, Thermo Fisher Scientific, USA), and spectral data were searched against the Uniprotkbbb Musculus proteome database. Consistent criteria (fold change ≥1.5 and p value < 0.05) and the KEGG pathway database (http://www.genome.jp/kegg/) and the GeneBench database (http://www.geneontology.org/) were used for further bioinformatic analysis.

### 2.28 Statistical analysis

Data were expressed as mean ± standard deviation (SD) and statistically analysed using GraphPad Prism 8.0. The unpaired *t*-test was used to determine statistical significance for single comparisons between the two groups. One-way analysis of variance (ANOVA) was used for multiple comparisons, and Tukey's test was used for pairwise comparisons between the two groups. *p* values less than 0.05 (*p* < 0.05) were considered statistically significant differences.

## 3 Results and discussion

### 3.1 Fabrication and characterization of PEKUU electrospinning fiber

**Fig. 1:**
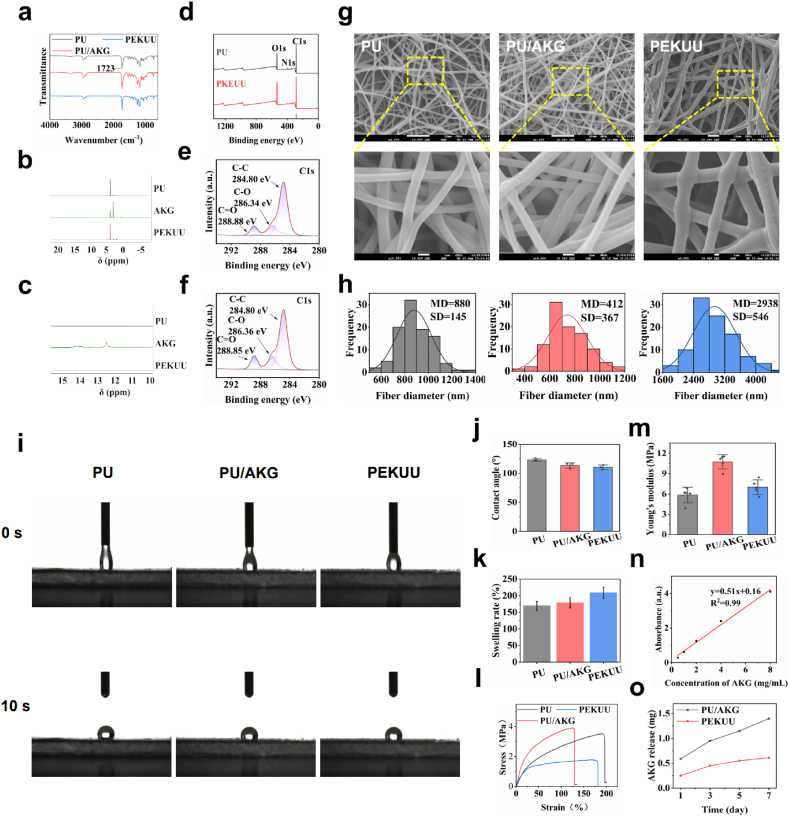
Morphological, physical, and mechanical properties of electrospinning fiber. (a) FTIR spectra of PU, PU/AKG, and PEKUU electrospinning fiber. (b) ^1^H NMR spectra of PU, AKG, PEKUU. (c) Local enlargement of ^1^H NMR spectra of PU, AKG, and PEKUU. (d) Full XPS spectrum of PU, PEKUU electrospinning fiber. (e) C1s of PU electrospinning fiber. (f) C1s of PEKUU electrospinning fiber. (g) SEM images of PU, PU/AKG, and PEKUU electrospinning fiber. (h) Diameter distribution of PU, PU/AKG, and PEKUU electrospinning fiber. (i) Water contact angle images of PU, PU/AKG, and PEKUU electrospinning fiber. (j) Water contact angle of PU, PU/AKG, PEKUU electrospinning fiber at 10s. (k) Dissolution rate of PU, PU/AKG, and PEKUU electrospinning fiber. Dissolution rate of PU, PU/AKG, PEKUU electrospinning fiber after 12h immersion in PBS solution. (l) Stress-strain curves of PU, PU/AKG, and PEKUU electrospinning fiber. (m) Young's modulus of PU, PU/AKG, and PEKUU electrospinning fiber. (n) Standard curve of AKG. (o) Results of AKG release from PU/AKG and PEKUU electrospinning fiber (in pH 7.4 PBS solution) over 7 days.

To achieve slowly release of AKG, the PEKUU fibers were synthesized, which was schematically described in Fig. S1. Firstly, poly(ε-caprolactone) (PCL) was esterified with AKG to form PCL-AKG prepolymer, followed by stepwise polycondensation using hexamethylene diisocyanate (HDI) as a chain extender to construct the polymeric backbone. Then the secondary chain extension with 1, 4-butanediol (BDO) was conducted to achieve linear architecture. At last, the PEKUU fibers were prepared through electrospinning technique for further wound dressing application. Also pure polyurethane (PU) and PU/AKG mixture were fabricated as comparison. In order to verify the synthetic success, various techniques were applied to prove that. Fig. 1a identified there were urethane carbonyl vibrations (∼1723 cm^−1^) in all samples. PU/AKG exhibited a red-shifted peak (1724 cm^−1^) with enhanced intensity, indicating hydrogen-bonding interactions between AKG carboxyl groups and urethane N-H. While for PEKUU, there was a distinct ester-linked C=O stretch at 1650 cm^−1^and a blue-shifted, intensified urethane peak at 1721 cm^−1^, collectively verifying covalent integration of AKG. As shown in Fig. 1b–c, H Nuclear Magnetic Resonance Spectra (^1^H NMR) confirmed the covalent bonding of AKG in PEKUU. The carboxylic acid proton resonance disappeared at δ12.2 ppm, and a characteristic β-methylene proton peak (-OOC-CH_2_-C(O)-) appeared at δ3.89 ppm, proving the formation of an ester bond. X-ray photoelectron spectroscopy (XPS) analysis in Fig. 1d demonstrated significantly elevated oxygen content in PEKUU (O/C atomic ratio 0.32 vs. 0.29 for PU), confirming esterification-mediated oxygen enrichment. The N1s peak shift from 399.8 to 400.0 eV indicated altered chemical environments of urethane linkages due to crosslinking. The C1s spectra in Fig. 1e–f further showed a 26 % increase in C=O bond contribution (288.85 eV) compared to PU, providing definitive evidence for covalent esterification.

In order to observe the morphology of prepared electrospinning fibers, scanning electron microscopy (SEM) was conducted. As shown in Fig. 1g and h, PU fibers possessed smooth surfaces with narrow diameter distribution (880 ± 145 nm), confirming electrospinning homogeneity. AKG-blended PU/AKG fibers showed significantly reduced diameters (739 ± 157 nm) with heterogeneous roughness attributed to the hydrogen bond interaction between AKG and PU. Whereas PEKUU fibers exhibited dramatically increased diameters (2938 ± 546 nm) and broad polydispersity, consistent with the low degree of polymerization and molecular weights (7.8 × 10^4^ Da) (Fig. S2). Hydrophilicity assessment (Fig. 1i) revealed persistent hydrophobicity in PU fibers (stable initial contact angle 123.5° over 10 s), while PU/AKG and PEKUU displayed time-dependent hydrophilization (declining to ≈ 110°) due to moisture absorption by free AKG carboxyl groups (PU/AKG) and capillary effects from roughened surfaces (PEKUU). Quantitative wettability hierarchy at 10 s was PU (123.5 ± 6.0°) > PU/AKG (113.4 ± 4.7°) > PEKUU (110.6 ± 3.9°) (Fig. 1j). Swelling kinetics in Fig. 1k showed PEKUU achieved the highest swelling ratio (214.65 ± 5.2 %), significantly surpassing PU/AKG (173.31 ± 3.1 %) and pure PU (169.38 ± 4.0 %), attributed to synergistic effects of chemical crosslink exposure and ester hydrolysis-generated hydrophilic domains. Mechanical testing results (Fig. 1l–m) demonstrated that PU/AKG achieved maximum Young's modulus (10.74 ± 1.0 MPa) from AKG-PU hydrogen bonding, yet minimal elongation at break (129.5 %). Conversely, PEKUU exhibited moderate modulus (7.01 ± 1.0 MPa) with enhanced extensibility (178.9 %), resulting from flexible segment incorporation restricting chain breaking. AKG release kinetics in Fig. 1n and o proved that PU/AKG exhibited burst release (0.59 ± 0.03 mg within 24 h, cumulative 1.40 ± 0.1 mg by 7 days), which might cause cellular acidosis. While equivalent PEKUU fibers released only 0.61 ± 0.05 mg cumulatively after 7 days, demonstrating chemical conjugation strategy enabled controlled liberation through ester hydrolysis.

### 3.2 *In vitro* Cytocompatibility, blood compatibility, and migration promoting capacity of PEKUU electrospinning fiber

**Fig. 2:**
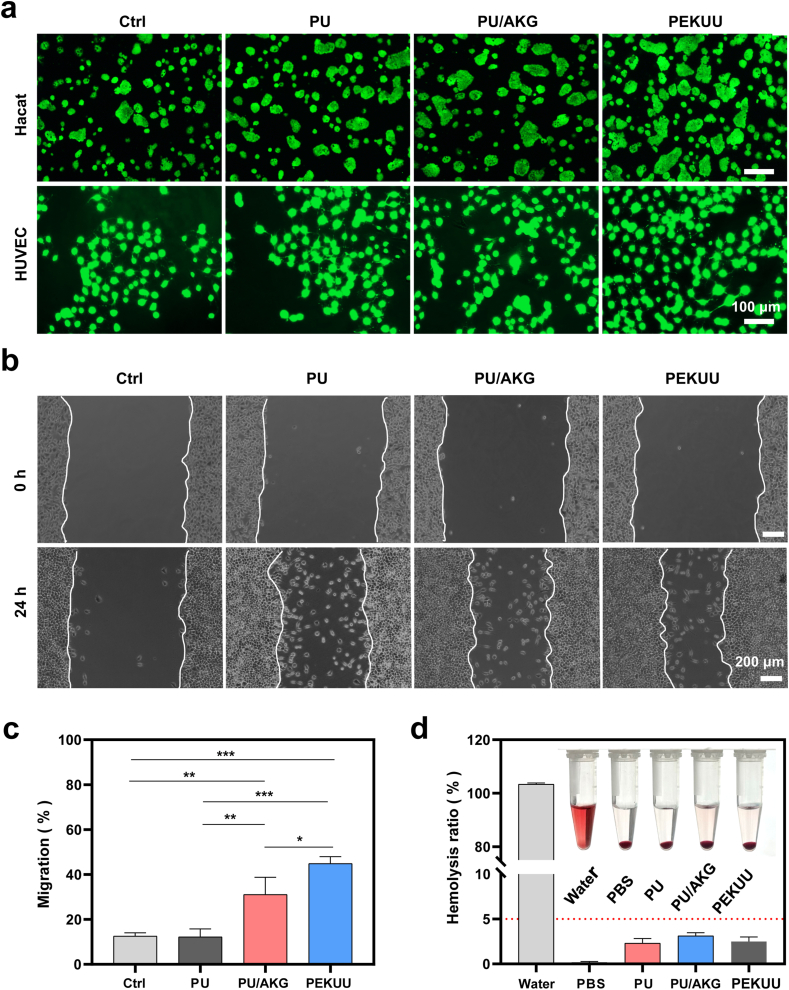
Cytocompatibility, blood compatibility, and pro-migration ability of electrospinning fiber. (a) Live/dead cell staining images of Hacat and HUVEC cells after co-incubation with pure medium and PU, PU/AKG, and PEKUU electrospinning fibers on the 3rd day. (b) The migration capability of L929 cells co-incubated with PU, PU/AKG, and PEKUU electrospinning after 24h. (c) Migration ratio of Hacat cells in the Ctrl, PU, PU/AKG, and PEKUU groups (n = 3). (d) Hemolysis ratios of various electrospinning (n = 3). *p* < 0.05 (“∗”), *p* < 0.05 (“∗”), *p* < 0.01 (“∗∗”), *p* < 0.001 (“∗∗∗”) and *p* < 0.0001 (“∗∗∗∗”).

Biocompatibility assessment is conducted as a prerequisite evaluation for wound dressing applications. The TCA cycle serves as the central hub integrating carbohydrate, lipid, and protein metabolism, with AKG recognized as a critical metabolic intermediate in this pathway [8]. Though physiologically relevant AKG concentrations were reported to stimulate cellular proliferation, excessive levels demonstrated cytotoxic effects through extracellular pH alteration. To systematically evaluate material safety, cytotoxic and proliferative responses of Hacat keratinocytes and HUVECs were examined following exposure to PU, PU/AKG, and PEKUU electrospinning fibers. Biocompatibility evaluation was systematically performed through dose-dependent cytotoxicity screening. CCK-8 assays quantitatively evaluated electrospinning fiber cytotoxicity across 24–72 h intervals. Elevating AKG concentrations to 3.2 μM significantly reduced cellular viability in both Hacat keratinocytes and HUVECs over 24–72 h exposure periods (Fig. S3). PU demonstrated excellent biocompatibility at all tested concentrations, confirming their suitability as inert biomaterial carriers. Both PU/AKG and PEKUU electrospinning fiber maintained cellular viability without observable toxicity till reaching the concentration of 3.2 μM. Based on release kinetic calculations, electrospinning sustaining 1.6 μM AKG release were selected as the optimal formulation for subsequent experimental evaluations. Fluorescence microscopy of Hacat and HUVEC cells in Fig. 2a revealed enhanced proliferative activity after PEKUU treatment compared to other groups. Quantitative analysis further supported that PEKUU-mediated AKG delivery significantly promoted cell proliferation without inducing morphological abnormalities. Collectively, PEKUU electrospinning fiber induced negligible cytotoxic effects on Hacat and HUVEC even after 72 h culture periods. Hemocompatibility and pro-migratory capacity were systematically evaluated as critical safety and functional parameters. Hemolysis assays demonstrated that PU, PU/AKG, and PEKUU electrospinning fiber exhibited hemolysis rates ranging from 2.1 % to 3.2 %, all remaining below the 5 % biocompatibility threshold (Fig. 2d). Furthermore, L929 fibroblast migration assays in Fig. 2b and c revealed PEKUU could significantly enhance cellular motility. Quantitative analysis showed PEKUU treated groups achieved 44.89 % migration rate, markedly surpassing Ctrl (12.53 %) and PU groups (12.28 %). These collective findings confirmed PEKUU's good biocompatibility and pro-migration capacity, fulfilling the essential requirements for clinical-grade wound dressings.

### 3.3 *In vitro* angiogenesis and ROS scavenging of PEKUU electrospinning fiber

**Fig. 3:**
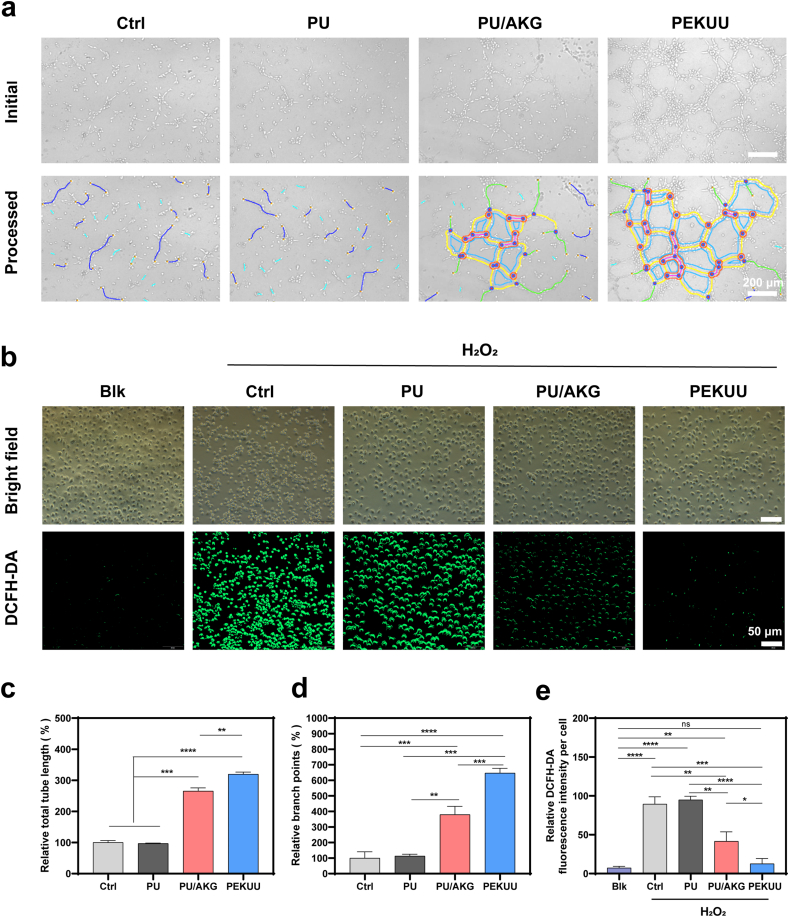
Angiogenesis and ROS scavenging property. (a) Examine angiogenesis properties in the Ctrl, PU, PU/AKG, and PEKUU groups (n = 3). (b) ROS scavenging property in the Ctrl, PU, PU/AKG, and PEKUU groups (n = 3). (c) Statistical analysis of Relative tube length which assesses the property of angiogenesis in the Ctrl, PU, PU/AKG, and PEKUU groups (n = 3), corresponding to (a). (d) Statistical analysis of Relative branch points which assess the property of angiogenesis in the Ctrl, PU, PU/AKG, and PEKUU groups (n = 3), corresponding to (a). (e) Statistical analysis of ROS scavenging property in the Ctrl, PU, PU/AKG, and PEKUU groups(n = 3), corresponding to (b). *p* < 0.05 (“∗”), *p* < 0.05 (“∗”), *p* < 0.01 (“∗∗”), *p* < 0.001 (“∗∗∗”) and *p* < 0.0001 (“∗∗∗∗”).

AKG serves as a pivotal intermediate metabolite in the TCA cycle, modulating intracellular metabolic pathways to supply energy and biosynthetic precursors essential for cellular functions [30]. During angiogenesis, AKG-supported energy metabolism is demonstrated to enhance endothelial cell proliferation and migration, thereby facilitating neovascularization through improved vascular network formation [13,40]. To validate that, a transwell co-culture system was employed to assess angiogenic potential using HUVECs exposed to PU, PU/AKG, and PEKUU (Fig. 3a). Quantitative morphometric analysis in Fig. 3c and d revealed that PEKUU-treated groups exhibited statistically significant enhancements in total vascular length and network branching compared to Ctrl, PU, and PU/AKG groups. The findings *in vitro* conclusively demonstrated the good pro-angiogenic capacity of PEKUU.

Macrophages exhibited remarkable heterogeneity and phenotypic plasticity, typically polarizing into pro-inflammatory M1 or pro-reparative M2 subtypes under microenvironmental cues [41]. In burn wounds, deep tissue damage and vascular disruption created hypoxic conditions that drove metabolic reprogramming in macrophages shifting from oxygen-dependent OXPHOS to glycolysis to adapt to oxygen deprivation [42,43]. The glycolytic dominance was demonstrated to suppress M2-associated OXPHOS and FAO pathways [44], thereby trapping macrophages in a persistent M1-polarized state. Sustained M1 activation led to excessive nitric oxide (NO) production via inducible nitric oxide synthase (iNOS), further generating reactive oxygen species (ROS) that perpetuated inflammatory responses and impaired healing [[45], [46], [47]]. To address this pathophysiology, the ROS-scavenging capacity of PEKUU electrospinning fiber was systematically evaluated. The therapeutic mechanism involved AKG supplementation to restore TCA cycle functionality, leveraging its carboxyl groups for reducing properties and epigenetic modulation capacity to neutralize ROS [[48], [49], [50]]. An oxidative stress model was established by stimulating bone marrow-derived macrophages (BMDMs) with H_2_O_2_, followed by intracellular ROS quantification using 2′,7′-dichlorodihydrofluorescein diacetate (DCFH-DA) probes. As shown in Fig. 3b, the fluorescence intensity of Ctrl group is stronger than that of the other group, which indicated the successful oxidative stress model. After PEKUU treatment, the fluorescence decreased mostly due to the anti-oxidative capability of released AKG.

### 3.4 *In vitro* Macrophage phenotype and bioenergetic metabolism regulation of PEKUU electrospinning fiber

**Fig. 4:**
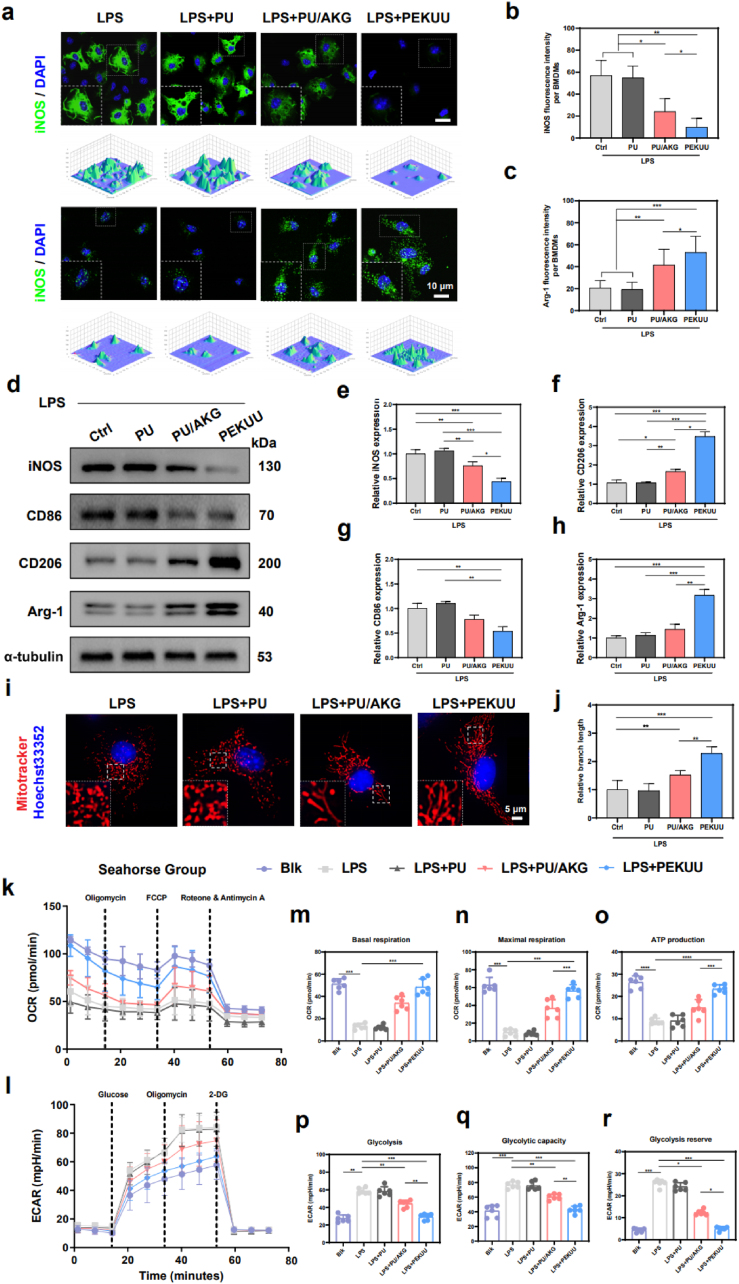
Effects of regulation phenotypes and bioenergetic metabolism model of macrophages. (a) Immunofluorescence images of BMDMs from the Ctrl, PU, PU/AKG, and PEKUU groups. (b) fluorescence intensity of iNOS expression in the Ctrl, PU, PU/AKG, and PEKUU groups (n = 3). (c) fluorescence intensity of Arg-1 expression in the Ctrl, PU, PU/AKG, and PEKUU groups (n = 3). (d) Western blotting images of CD86, iNOS, CD206, Arg-1, and Tubulin in the Ctrl, PU, PU/AKG, and PEKUU groups. (e) Relative expression of iNOS, (f) CD206, (g) CD86, and (h) Arg-1 in the Ctrl, PU, PU/AKG, and PEKUU groups (n = 3). (i)Mitochondria immunofluorescence images of BMDMs with LPS from the Ctrl, PU, PU/AKG, and PEKUU groups (j) Statistical analysis of mitochondria length of BMDMs. (k) Oxygen consumption rate (OCR)-Time curve of Raw264.7 in Blk, LPS, LPS + PU, LPS + PU/AKG, and LPS + PEKUU groups (n = 6). (l) Extracellular acidification rate (ECAR)-Time curve of Raw264.7 in Blk, LPS, LPS + PU, LPS + PU/AKG, and LPS + PEKUU groups (n = 6). (m) Mitochondrial basal respiration, (n) maximal respiration, and (o) ATP production of Raw264.7 tested by Seahorse energy detection system in different groups (n = 6). (p) Glycolysis, (q) glycolytic capacity, and (r) glycolysis reserve of Raw264.7 tested by Seahorse energy detection system in different groups (n = 6). *p* < 0.05 (“∗”), *p* < 0.01 (“∗∗”), *p* < 0.001 (“∗∗∗”) and *p* < 0.0001 (“∗∗∗∗”).

Macrophages are the major immune cells in skin wounds and play an important role in the process of wound repair [51], which can be categorized into pro-inflammatory M1-type macrophages and anti-inflammatory M2-type macrophages. M1-type macrophages can secrete pro-inflammatory cytokines, such as TNF-α and IL-6 [52], whereas M2-type macrophages secrete anti-inflammatory cytokines, such as IL-10. Though M1-type macrophages can remove and phagocytose pathogens to achieve anti-inflammatory effects, burns lead to overactivation of M1-type macrophages, resulting in the sustained release of inflammatory factors such as IL-6 and TNF-α to exacerbate inflammatory responses. Then delayed collagen deposition impedes wound healing [48]. Therefore, switching the macrophage polarization phenotype is critical for promoting burn wound healing. To assess the immunomodulatory capacity of the prepared electrospinning fibers, LPS-activated BMDMs were co-cultured with various fiber groups. Morphological analysis revealed that BMDMs in the PEKUU group exhibited distinct M2-polarized characteristics compared to other groups (Fig. S5). Subsequently, fluorescence imaging in Fig. 4a demonstrated that the PEKUU group showed significantly reduced expression of the M1 marker iNOS and markedly increased expression of the M2 marker Arginase-1 (Arg-1) compared to the Ctrl, PU, and PU/AKG groups. Statistical analysis revealed that the iNOS fluorescence intensity in the PU/AKG and PEKUU groups decreased to 24.08 % and 12.87 %, respectively, compared to the Ctrl and PU groups (Fig. 4b). Conversely, the Arg-1 relative fluorescence intensity in the PU/AKG and PEKUU groups increased to 41.49 % and 52.95 % respectively, compared to the Ctrl and PU groups (Fig. 4c). Those results collectively demonstrated that the PEKUU electrospinning fiber exhibited immunomodulatory capabilities to reprogram LPS-activated the M1-polarized pro-inflammatory macrophages toward the M2-polarized anti-inflammatory phenotype. To further validate the immunomodulatory effects of PEKUU and its role in bioenergetic metabolic reprogramming, Western blot (WB) analysis was performed to evaluate the phenotypic polarization. The WB results showed that the PEKUU group exhibited the most pronounced reduction in M1 markers (iNOS and CD86) and the highest elevation in M2 markers (Arg-1 and CD206) compared to the other groups (Fig. 4e–h). To investigate the immunomodulatory effects of PEKUU on BMDMs at the transcriptional level, quantitative reverse transcription polymerase chain reaction (RT-qPCR) was conducted. Statistical analysis revealed that the PEKUU group showed 75 % and 60 % reductions in M1 markers (iNOS and CD86, respectively) compared to Ctrl group (Fig. S8). While M2 markers (Arg-1 and CD206) were upregulated about 200 % and 400 %, respectively (Fig. S8). The above results confirmed the modulating capability of PEKUU in BMDM phenotypic polarization.

TCA cycle serves as the central pathway for complete oxidation of acetyl-CoA into water and carbon dioxide with concomitant energy production [12]. Proper cellular energetic in macrophage requires intact mitochondrial function. Previous studies demonstrated that macrophages under inflammatory conditions exhibited mitochondrial dynamics imbalance, leading to functional impairment [53], then reducing ATP production [54]. As a critical TCA cycle intermediate, AKG was metabolized to succinyl-CoA, which facilitated electron transport chain (ETC) activity and enhanced OXPHOS efficiency, thereby promoting ATP generation [55]. Based on that, we hypothesized that PEKUU might restore mitochondrial function in macrophage by sustaining AKG release, thereby enhancing OXPHOS to promote ATP generation. To validate this hypothesis, viable BMDMs were treated with LPS and various fibers, followed by live-cell mitochondrial labeling using MitoTracker. As shown in Fig. 4i, LPS-stimulated macrophages exhibited mitochondrial hyperfission, while PEKUU fiber treatment progressively attenuated mitochondrial fragmentation and restored network homeostasis. Quantitative analysis revealed that the PEKUU group significantly restored mitochondrial branch length compared to that of Ctrl, PU, and PU/AKG groups, demonstrating the capacity in regulating mitochondrial dynamics and restoring the mitochondrial network.

Subsequently, Seahorse energy detection system was applied to test the OXPHOS level after various treatments. The statistical results in Fig. 4k–n showed that the basal, maximal mitochondrial respiration and ATP production levels in the PEKUU group were higher than those in Ctrl, PU and PU/AKG groups, which proved that the PEKUU greatly enhanced the mitochondrial OXPHOS levels in macrophages. Meanwhile, Elisa assay also confirmed that the ATP was restored by about 50 % after PEKUU treatment of LPS-activated M1 (Fig. S6a). Subsequently, the glycolysis levels of various groups were evaluated. As shown in Fig. 4o–r, the glycolysis level, glycolytic capacity and reserve were all significantly decreased to reduce inflammation response. As previous reported, macrophages stimulated by inflammatory factors developed mitochondrial dysfunction and exhibited two metabolic discontinuities in the TCA cycle [56]. Both IDH and SDH functioned as crucial rate-limiting enzymes in the TCA cycle [57,58] to govern overall bioenergetic efficiency. Then the enzymatic activities of IDH and SDH were tested by enzyme-linked ELISA kit. As shown in Fig. S6, LPS treatment significantly reduced the activities of IDH and SDH. After PEKUU treatment, the enzymatic acivities of them were enhaced, which meant the TCA cycle was restored to produce ATP. The above data indicated that the slowly released AKG from PEKUU electrospinning fibers showed potential capability to reduce glycolysis and promote mitochondrial OXPHOS and ATP production, which was beneficial for wound healing.

### 3.5 *In vivo* promotion of burn wound healing via PEKUU electrospinning fiber

**Fig. 5:**
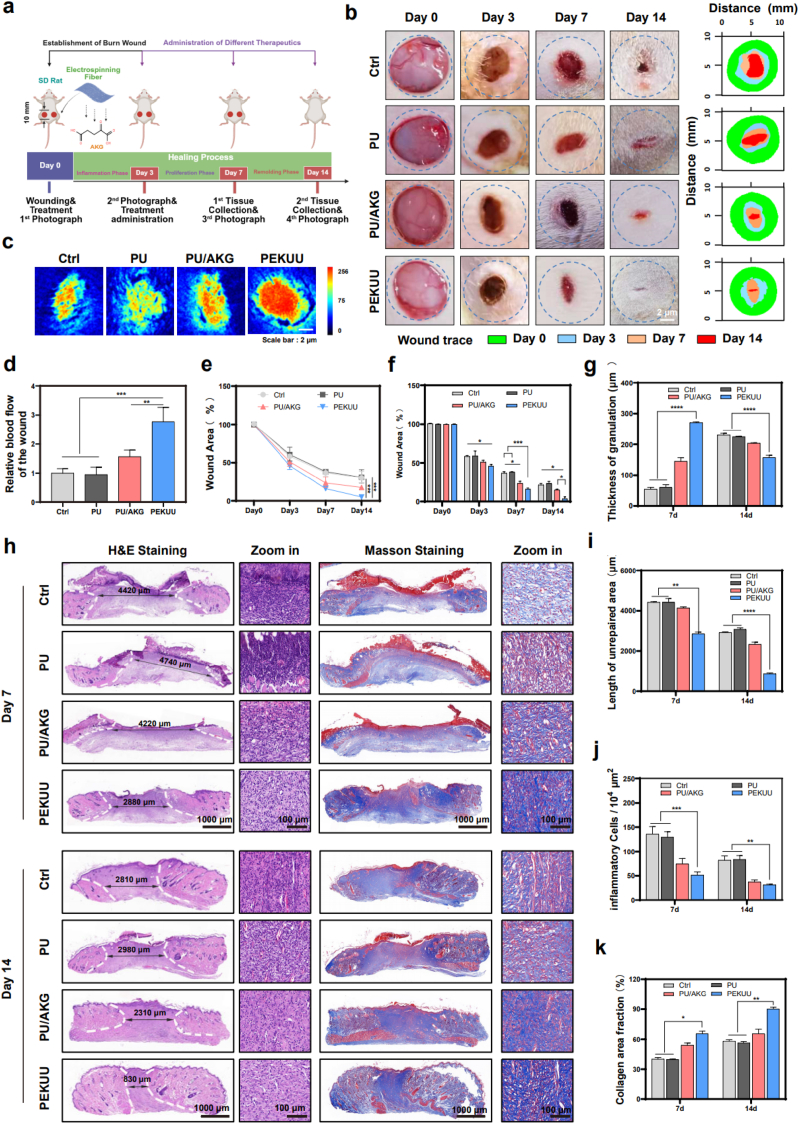
Burn wound healing. (a) Schematic of the *in vivo* study design. (b) Representative images of burn wounds in various treatment groups. (c) Image of blood flow supply to the burn wounds in various treatment groups. (n = 3). (d) Statistical analysis of blood flow supply to the burn wounds in various treatment groups. (n = 3), corresponding to (c). (e) Quantitative analysis of wound area (n = 5). (f) Quantitative analysis of wound area, corresponding to (e). (n = 5) (g) Statistical analysis of the thickness of granulation. (h) H&E and Masson staining of burn wounds on day 7 and 14 (n = 3). (i) Statistical analysis of unrepaired area length. (j) Statistical analysis of inflammatory cells. (k) Statistical analysis of collagen deposition of the wound area. (n = 3). *p* < 0.05 (“∗”), *p* < 0.01 (“∗∗”), *p* < 0.001 (“∗∗∗”) and *p* < 0.0001 (“∗∗∗∗”).

The PEKUU electrospinning fiber demonstrated good performance *in vitro* assessments including biocompatibility, anti-inflammatory and antioxidant activities, immune regulation, and energy metabolism modulation, fulfilling the criteria for ideal wound dressings. Subsequent validation experiments were conducted to evaluate the *in vivo* effect of PEKUU on burn wound. A deep second-degree scald injury model was established on the dorsal skin of SD rats, followed by different treatments and photographic documentation on days 3, 7, and 14. Rats were sacrificed on days 7 and 14 for histological analysis of collected wound tissues, which was schematically shown in Fig. 5a. As illustrated in Fig. 5b, the PEKUU group exhibited remarkable wound size reduction on days 3 and 7 compared to other groups. Infrared blood flow imaging on day 7 (Fig. 5c) revealed that the PEKUU group demonstrated approximately threefold higher blood perfusion recovery compared to Ctrl group (Fig. 5d), suggesting enhanced angiogenic potential. By the day 14, complete eschar elimination and visible epidermal regeneration were observed in PEKUU treated wound. Quantitative analysis confirmed statistically significant wound area reduction in the PEKUU group compared to other interventions (Fig. 5e). These collective findings demonstrated that PEKUU electrospinning fiber significantly accelerated burn wound healing.

To further investigate the burn wound-healing effect of PEKUU, tissue sections were stained and analysed using histological techniques to evaluate repair outcomes. Hematoxylin and eosin (H&E) staining was employed to compare differences in wound healing across experimental groups. On day 7, inflammatory cell infiltration, unhealed areas, and granulation tissue regeneration were assessed as primary evaluation criteria. Quantitative analysis of granulation tissue thickness, a critical indicator of wound repair, revealed that the PEKUU group exhibited approximately threefold greater relative granulation tissue thickness compared to the other groups (Fig. 5f and S12). Moreover, on day 7, the PEKUU group demonstrated the lowest unhealed area and inflammatory cell counts among all groups (Fig. 5h and i). Additionally, as shown in Fig. 5j, collagen content in PEKUU-treated wounds reached 63 %, while only 40 % was observed in Ctrl group. By day 14, unsaturated granulation tissue in the PEKUU group progressed to the re-epithelialization phase, with thickness reduced to 50 % of the Ctrl group, indicating entry into the terminal healing stage. Concurrently, inflammatory cell levels decreased to 32 % of the Ctrl (Fig. 5g and h), while collagen deposition increased to 90.2 % (Fig. 5j). The *in vivo* results demonstrated that the PEKUU electrospinning fiber accelerated deep second-degree burn healing by effectively resolving inflammation, promoting collagen synthesis, and enhancing tissue regeneration.

### 3.6 *In vivo* antioxidative, anti-inflammatory, and Macrophage phenotype regulation of PEKUU electrospinning fiber

**Fig. 6:**
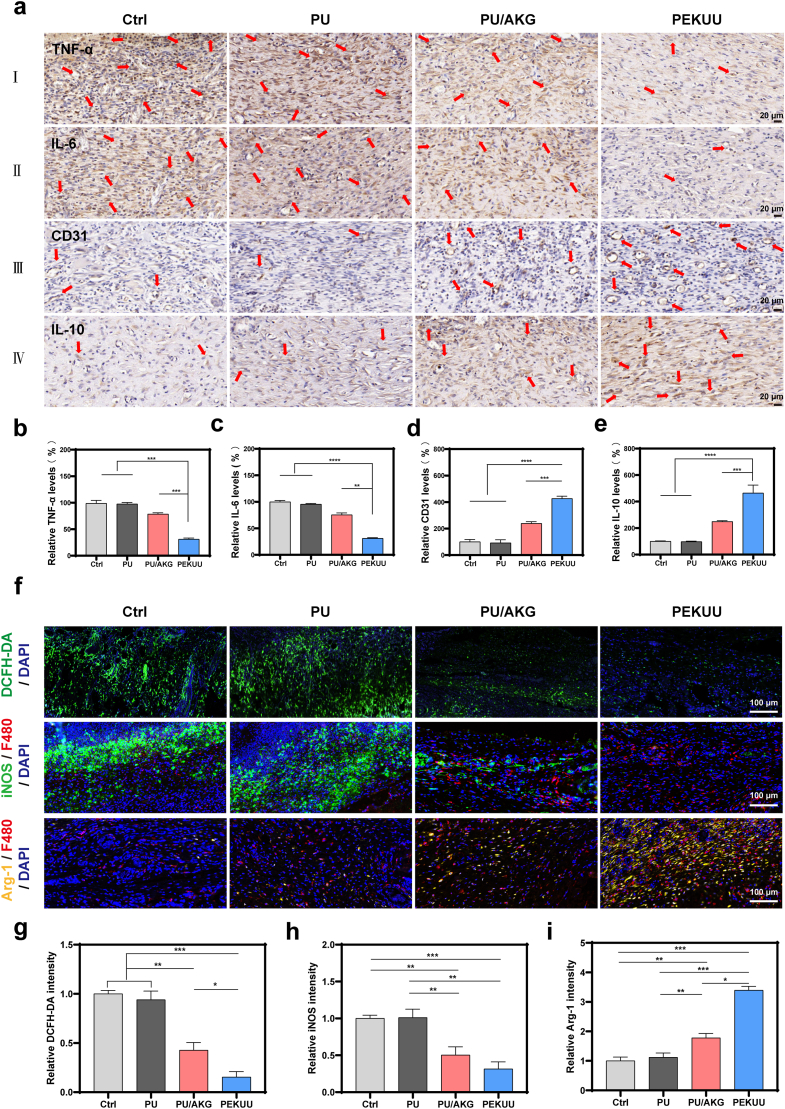
Immunohistochemistry and Immunofluorescence staining of burn wound tissues. (a) Immunohistochemistry staining of TNF-α (Ⅰ), IL-6 (Ⅱ), CD31 (Ⅲ), and IL-10 (Ⅳ) in the wound tissues from the Ctrl, PU, PU/AKG and PEKUU groups on the day 7 and day 14. (b) Positive expression ratios of CD31, (c) IL-10, (d) TNF-*α* and (e) IL-6, in the Ctrl, PU, PU/AKG and PEKUU groups (n = 3), corresponding to (a). (f) Immunofluorescence images of ROS, iNOS, and Arg-1 in the wound tissues from the Ctrl, PU, PU/AKG, and PEKUU groups. (g) Relative expression ratios of DCFH-DA, (h) iNOS, and (i) Arg-1 in the Ctrl, PU, PU/AKG and PEKUU groups (n = 3), corresponding to (f). *p* < 0.05 (“∗”), *p* < 0.01 (“∗∗”), *p* < 0.001 (“∗∗∗”) and *p* < 0.0001 (“∗∗∗∗”).

Macrophage polarization plays essential role in wound healing [58]. Previous studies reported that AKG derivate could promote M2 polarization through JMJD3-dependent epigenetic reprogramming to upregulate IL-4 expression [15], while concurrently suppressing hyper-inflammation via TET-mediated DNA demethylation and NF-κB signaling inhibition [59,60]. To validate AKG's anti-inflammatory efficacy, immunohistochemical analyses of TNF-α and IL-6 were performed. As shown in Fig. 6a–c, TNF-α and IL-6 levels in the PEKUU group were reduced to 57 % and 48 % respectively, compared to the Ctrl group. Mechanism investigation through CD31 and IL-10 immunohistochemistry revealed enhanced pro-angiogenic and anti-inflammatory effects in the PEKUU group. Fig. 6a and d demonstrated significantly greater CD31-positive areas in PEKUU-treated wounds, with expression levels twofold higher than the Ctrl group. IL-10, a hallmark cytokine of M2 macrophages with dual anti-inflammatory and pro-healing functions [35,61], exhibited 43 %, 41 %, and 19.8 % higher expression in the PEKUU group compared to Ctrl, PU, and PU/AKG groups, respectively (Fig. 6a and e). The *in vivo* evidence confirmed PEKUU showed multifunctional efficacy in suppressing pathological inflammation, enhancing vascularization, and promoting immunomodulation to accelerate deep second-degree burn healing.

To validate the ROS-scavenging capacity and macrophage phenotype-modulating effects of PEKUU in burn wounds, immunofluorescence staining for ROS and macrophage biomarkers was performed on frozen wound tissues. As previously described, extensive cellular damage and mitochondrial dysfunction in burn injuries led to electron leakage, generating superoxide anions that combined with free electrons to form ROS, which impeded wound repair [62]. Additionally, M1 macrophage-derived iNOS overproduced NO, further contributing to ROS accumulation [63]. Consequently, ROS clearance represented a critical therapeutic strategy. As demonstrated in Fig. 6f, the PEKUU group exhibited significantly lower ROS fluorescence intensity compared to the other groups. Quantitative analysis in Fig. 6g revealed the PEKUU group showed a marked ROS reduction to 23.8 %. The above results confirmed that PEKUU could effectively mitigate oxidative stress by scavenging ROS, thereby alleviating acute inflammatory and oxidative damage.

**Fig. 7:**
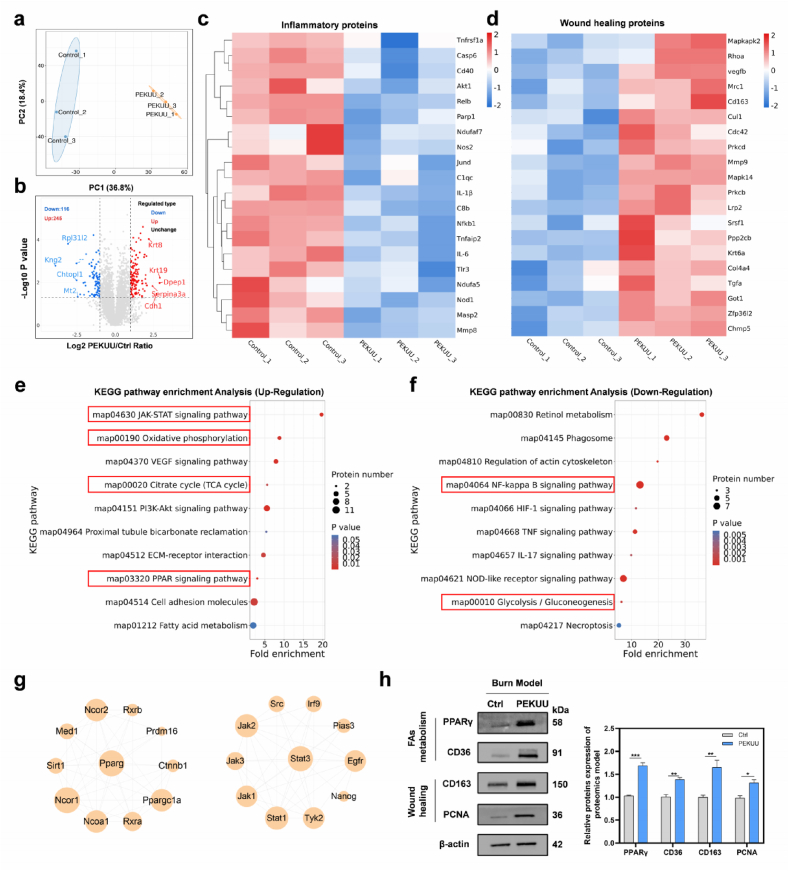
The proteomic in the burn rat model was treated by the PEKUU electrospinning fiber. (a) Principal component analysis (PCA) of differentially expressed proteins in the wound tissues of the Ctrl (No-treatment) and PEKUU (Exp) groups (n = 3). (b) Volcano plot showing upregulated and downregulated proteins after treatment with the PEKUU electrospinning. (c) Heat map of significant differentially expressed proteins of inflammation after treatment with the PEKUU electrospinning. (d) Heat map of significant differentially expressed proteins of wound healing after treatment with the PEKUU electrospinning. (fold change ≥1.5 and *p* < 0.05). (e) KEGG pathway enrichment analysis for differentially of up-regulation signaling pathways in PEKUU-treated wounds versus Ctrl wounds. (f) KEGG pathway enrichment analysis for differentially of down-regulation signaling pathways in PEKUU-treated wounds versus Ctrl wounds. (g) PPI of pro-healing protein of PPARγ and STAT3. (h) Western blot of FAs metabolism and wound healing protein of proteomics model, and its statistical analysis. *p* < 0.05 (“∗”), *p* < 0.01 (“∗∗”), *p* < 0.001 (“∗∗∗”) and *p* < 0.0001 (“∗∗∗∗”).

To investigate the *in vivo* immunomodulatory capacity of PEKUU, immunofluorescence co-staining was performed on wound sections. As shown in Fig. 7f, the PEKUU group exhibited substantially reduced iNOS expression compared to the Ctrl, PU, and PU/AKG groups. Quantitative analysis in Fig. 6h demonstrated that iNOS levels in the PEKUU group were reduced more than 4 times compared to the Ctrl group. Conversely, Fig. 7i revealed approximately threefold higher expression of Arg-1 in the PEKUU group. The results indicated that PEKUU could mediate macrophage polarization, which was attributed to the sustained release of AKG to orchestrate anti-inflammatory, antioxidant, pro-reparative, and immunomodulatory actions in burn wounds.

### 3.7 Analysis of the proteomic of burn skin after PEKUU treatment

To elucidate the mechanism underlying the accelerated deep second-degree burn wound repair mediated by PEKUU, proteomic profiling was conducted on wound tissues after treatment. As shown in Fig. 7a, PEKUU treated wound tissues exhibited marked heterogeneity compared to Ctrl group, with high intra-group consistency among biological replicates. Volcano plot analysis identified 361 differentially expressed proteins between groups, including 245 upregulated and 116 downregulated proteins (Fig. 7b). Heatmap visualization confirmed pronounced intergroup disparities in protein expression profiles (Fig. 7c). Notably, PEKUU treated tissues demonstrated significant downregulation of inflammation-associated proteins, alongside robust upregulation of pro-healing proteins, including CD163, which was a canonical M2 macrophage marker (Fig. 7d). Kyoto Encyclopedia of Genes and Genomes (KEGG) pathway analysis revealed that PEKUU upregulated OXPHOS signaling, TCA cycle, and PPAR-mediated fatty acid metabolism pathways (Fig. 7e), which was mechanistically linked to M2 macrophage polarization. As well known, OXPHOS and FAO represent the primary energy metabolism pathways in M2 macrophages [48]. Under IL-4 or IL-13 stimulation, M2 macrophages utilize FAO to generate ATP and mediate tissue repair and anti-inflammatory responses [64]. In deep burn injuries, severe vascular disruption and hypoxia drive macrophage to reprogram its metabolic preference toward glycolysis, a faster but less efficient energy-producing pathway. Glycolysis and FAO exhibit reciprocal regulatory roles in macrophage polarization [65]. As shown in Fig. 7f, PEKUU downregulated glycolysis and pro-inflammatory NF-κB signaling. As previously noted, macrophage metabolic programs exhibited reciprocal regulation [66,67], and the observed suppression of glycolysis in KEGG analysis confirmed the enhanced OXPHOS and FAO in PEKUU-treated wounds.

The downregulation of NF-κB signaling provided indirect evidence of macrophage phenotypic switching from pro-inflammatory M1 to reparative M2 dominance. Clustering analysis further revealed enhanced biological processes related to skin development (Fig. S13), indicating PEKUU facilitated cutaneous regeneration. As reported, Jak-stat pathway played an important role in wound healing, and STAT3 could promote the polarization of M2 macrophage [68]. Protein-Protein Interactin (PPI) analysis of PPARγ and STAT3 in Fig. 7g, it predicted which proteins PPARγ and STAT3 might interact with to promote burn wound repair, which orchestrated downstream protein alterations critical to metabolic and reparative processes. Subsequently we selected a number of classic FAO metabolism and wound healing proteins based on previous proteomics data for validation by western blot. Results in Fig. 7h confirmed that FAO metabolic pathways and wound repair mechanisms were significantly upregulated in PEKUU-treated tissues. These coordinated metabolic and immunological reprogramming events synergistically accelerated wound closure, confirming PEKUU's multifunctional therapeutic efficacy in deep second-degree burn repair.

### 3.8 Study of healing mechanism of PEKUU electrospinning fiber

**Fig. 8:**
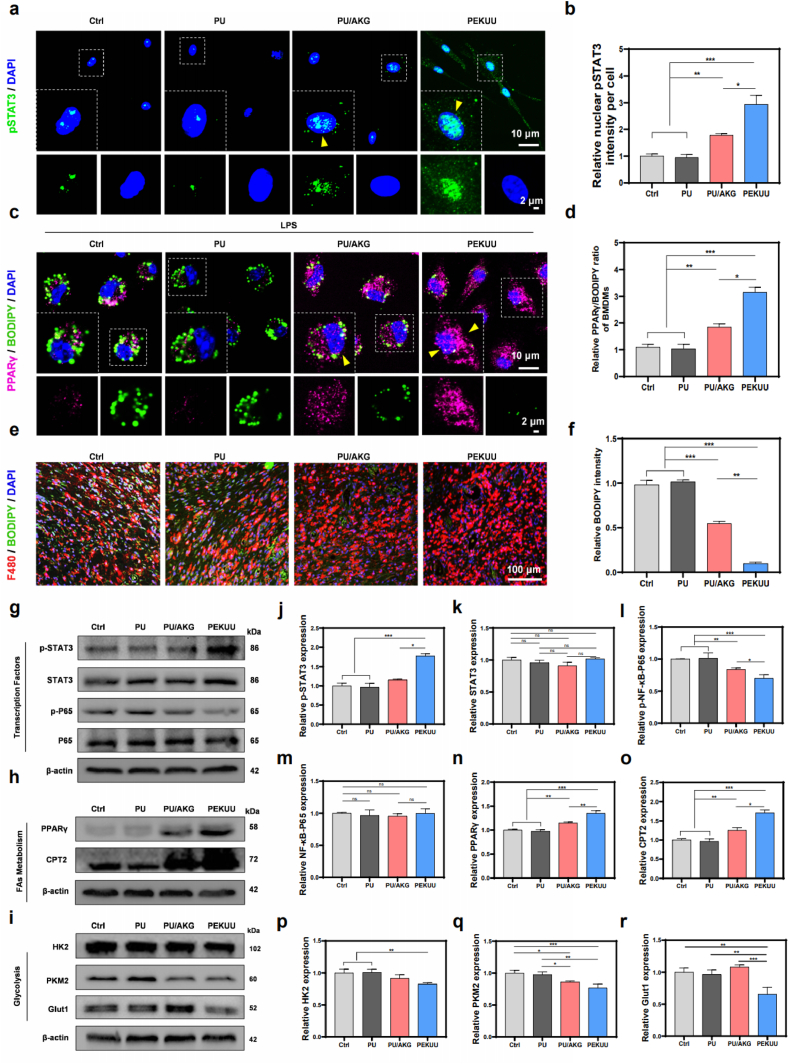
Pro-healing mechanism of the PEKUU electrospinning fiber. (a) Immunofluorescence images of pSTAT3 nuclear translocation in BMDMs (n = 5). The yellow arrows point to nuclear translocated pSTAT3 transcription factor. (b) Statistical analysis of BMDMs pSTAT3 nuclear translocation levels. (c) Immunofluorescence images of PPARγ and BODIPY in BMDMs (n = 5). The yellow arrows point to nuclear translocated PPARγ transcription factor. (d) Statistical analysis of BMDMs PPARγ and BODIPY levels. (e) Immunofluorescence images of BODIPY levels in burn wound tissue. (n = 3). (f) Statistical analysis of burn wound tissue BODIPY levels. (g) Protein expression of pSTAT3, STAT3, p-NF-κB-P65, and NF-κB-P65 in BMDMs after treated with LPS and various electrospinning fibers analysed by western blot (n = 3). (h) Protein expression of PPARγ and CPT2 in BMDMs after treated with LPS and various electrospinning fibers analysed by western blot. (i) Protein expression of HK2, PKM2, and Glut1 in BMDMs after treated with LPS and various electrospinning fibers analysed by western blot (n = 3). (j) Statistical analysis of STAT3, (k) p-NF-κB-P65, (l) NF-κB-P65 and (m) p-NF-κB-P65 in the Ctrl, PU, PU/AKG, and PEKUU groups (n = 3), corresponding to (g). (n) Statistical analysis of PPARγ and (o) CPT2 in the Ctrl, PU, PU/AKG, and PEKUU groups (n = 3), corresponding to (h). (p) Statistical analysis of HK2, (q) PKM2, and (r) Glut1 in the Ctrl, PU, PU/AKG, and PEKUU groups (n = 3), corresponding to (i). *p* < 0.05 (“∗”), *p* < 0.05 (“∗”), *p* < 0.01 (“∗∗”), *p* < 0.001 (“∗∗∗”) and *p* < 0.0001 (“∗∗∗∗”). (For interpretation of the references to colour in this figure legend, the reader is referred to the Web version of this article.)

To investigate the mechanism of PEKUU electrospinning fiber to promote burn wound healing, proteomics analysis was conducted. KEGG pathway analysis showed that PEKUU significantly upregulated the PPAR lipid metabolism pathway [69], JAK-STAT pathway, OXPHOS pathway, and TCA cycle pathway in wound tissues. Those pathways were closely associated with PEKUU's promotion of burn wound healing. As well known, the PPAR pathway was a core pathway in lipid metabolism [70], closely associated with physiological processes such as fat mobilization and FAO [71]. FAO was a key factor in promoting M2-type macrophage polarization [72], and PPARγ was the main factor in the pathway [45]. Also the JAK-STAT pathway encompassed various physiological activities, including regulation of multiple cytokines and transcription factors associated with wound healing [73]. Based on the proteomics screening, we found that STAT3 protein expression was significantly increased after PEKUU treatment. Therefore, PPARγ and STAT3 might be the two key proteins response to PEKUU treatment. In order to prove that, p-STAT3 antibody was stained for immunofluorescence to confirm STAT3 expression. As shown in Fig. 8a and b, the level of p-STAT3 nuclear expression was much higher in the PEKUU group than that in Ctrl, PU, and PU/AKG groups. Further, WB was conducted to validate the above results. As shown in Fig. 8g–i, total STAT3 levels remained unchanged in the PEKUU group while phosphorylated STAT3 levels increased, which indicated that PEKUU upregulated the STAT3 protein in the JAK/STAT pathway. Based on the above results, it could be infered that PEKUU might promote burn repair by stimulation of STAT3.

As the inflammatory microenvironment of burn wounds could impair macrophage lipid processing capacity and weaken M2 polarization, it was necessary to investigate whether PEKUU could affect lipid accumulation through PPARγ under inflammatory conditions. As shown in Fig. S14, after LPS stimulation, the PPARγ expression in BMDMs was lower than that of blank group, accompanying with lipid accumulation. It was proved that the function of PPARγ was impaired and the biological process of lipid processing was inhibited under inflammation. As shown in Fig. 8c, after PEKUU treatment, the lipid accumulation was significantly reduced and the PPARγ expression in nucleus was increased. The above results revealed that the PEKUU could potentially modulate lipid processing capacity of BMDMs by PPARγ. Furthermore, *in vivo* evaluation was also conducted by lipid labeling on burn wound. As shown in Fig. 8e, after PEKUU treatment, the lipid accumulation was significantly reduced compared with the other groups. The above results indicated that the wound's lipid processing capacity was enhanced after PEKUU treatment, which aligned with the enhancement of FAO to promote M2 polarization.

To validate the accuracy of proteomics analysis, WB was used to examine the expression of FAO, glycolysis, and the inflammatory transcription factor NF-κB. The results in Fig. 8h showed that FAO-related proteins such as PPARγ and carnitine palmitoyltransferase 2 (CPT2) were significantly upregulated. While, Fig. 8i presented that glycolysis-related proteins such as glucose transporter 1 (GLUT1), pyruvate kinase M2 (PKM2), hexokinase 2 (HK2) were significantly downregulated. Additionally, Fig. 8g showed total NF-κB-P65 remained unchanged, while phosphorylated NF-κB-P65 was significantly downregulated. Those results sufficiently demonstrated that the proteomics results were scientifically reliable.

By the above results, the PEKUU improved the biological process of FAO in macrophages by slow release of AKG, thereby the phosphorylation level of STAT3 and the PPARγ expression were upregulated. With the enhancement of FAO, the metabolic pattern was regulated from glycolysis to OXPHOS to promote M2-type polarization. Furthermore, the PEKUU downregulated glycolysis and NF-κB-P65 phosphorylation levels to supress inflammation and M1 polarization. Then a microenvironment for wound regeneration was built for burn repair. The PEKUU electrospinning fiber showed potential capability to regulate macrophage polarization through mediating metabolic reprogramming. The type shift from M1 to M2 endowed macrophage anti-inflammatory, antioxidant, and energy supply functions to coordinate in multiple dimensions, ultimately accelerating burn wound healing.

## 4 Conclusion

In this study, a novel PEKUU electrospinning fiber electrospinning fiber was synthesized through *in-situ* polycondensation for sustained delivery of bioactive AKG to treat burn wounds. As a critical intermediate in the TCA cycle, AKG demonstrated significant regulatory effects on promoting skin cell proliferation, migration, and tube formation, while enhancing macrophage ROS scavenging and immunomodulatory functions. The PEKUU fiber could effectively address critical challenges in burn wound healing by reversing TCA cycle disruption-induced energy deficits in macrophages through AKG's anaplerotic effect, thereby ameliorating inflammatory infiltration within the wound microenvironment. Following sustained AKG release into the wound bed, continuous activation of cellular TCA cycle metabolism was observed, accompanied by metabolic reprogramming of macrophages from glycolysis to OXPHOS. The transition facilitated FAO-driven energy production in highly activated M2 macrophages, while AKG-mediated oxidative stress alleviation promoted phenotypic switching from pro-inflammatory M1 to anti-inflammatory M2 macrophages. Proteomic analyses conclusively demonstrated that the PEKUU exerted consistent anti-inflammatory and antioxidant effects through coordinated inhibition of NF-κB signaling and activation of both JAK-STAT and PPAR pathways, effectively modulating macrophage energy metabolism to drive M2 phenotypic polarization. Then accelerated burn repair was achieved. The investigation established a novel strategy for promoting burn wound healing through macrophage polarization via metabolic reprogramming, providing a promising therapeutic platform for clinical burn wound management.

## CRediT authorship contribution statement

**Haoyang Wu:** Writing – original draft, Methodology, Investigation, Formal analysis, Data curation. **Qimeng Wu:** Investigation, Formal analysis, Data curation. **Chen Liang:** Investigation, Data curation. **Jiali Hua:** Validation, Data curation. **Lingyi Meng:** Software, Formal analysis. **Paweł Nakielski:** Software, Methodology. **Chenyan Lu:** Data curation. **Filippo Pierini:** Writing – review & editing. **Liqun Xu:** Writing – review & editing. **Yunlong Yu:** Writing – review & editing, Writing – original draft, Supervision, Funding acquisition, Conceptualization. **Qianqian Luo:** Writing – review & editing, Supervision, Funding acquisition.

## Author information

All authors have approved the final version of this manuscript.

## Ethics approval and consent to participate

All animal treatment procedures were conducted according to the Chinese Animal Management Rules of the Ministry of Health and were authorized by the Animal Ethics Committees of Nantong University research program protocol (S20250915-005) and performed in accordance with the Guide for the Care and Use of Laboratory Animals.

## Declaration of competing interest

The authors declare that they have no known competing financial interests or personal relationships that could have appeared to influence the work reported in this paper.

## Data Availability

The data that support the findings of this study are available from the corresponding author upon reasonable request.
