# Supplementary material for: Immunoregulatory electrospinning fiber mediates Macrophage energy metabolism reprogramming to promote burn wound healing
Source: Mater Today Bio. 2025 Oct 16;35:102430. doi: 10.1016/j.mtbio.2025.102430 (PMC12593653; doi:10.1016/j.mtbio.2025.102430)
Supplement: Multimedia component 1 [file mmc1.docx]

**Supplementary Materials**

**Immunoregulatory Electrospinning Fiber Mediates Macrophage Energy Metabolism Reprogramming to Promote Burn Wound Healing**

**Haoyang Wu^1#^, Qimeng Wu^2,3#^, Chen Liang^4^, Jiali Hua^1^, Lingyi Meng^1^, Paweł Nakielski^5^, Chenyan Lu^1^, Filippo Pierini^5^, Liqun Xu^3^, Yunlong Yu^2*^, Qianqian Luo^1*^**

^1^ Department of Hypoxic Biomedicine, Institute of Special Environmental Medicine and Coinnovation Center of Neuroregeneration, Nantong University, 226019 Nantong, P.R. China

^2^ Institute of Burn Research, Southwest Hospital, Third Military Medical University (Army Medical University), 400038 Chongqing, P.R. China

^3^ BRICS Joint Laboratory on Biomedical Materials, School of Materials and Energy, Southwest University, 400715 Chongqing, P. R. China

^4^ Multidisciplinary Centre for Advanced Materials, Institute for Frontier Medical Technology, School of Chemistry and Chemical Engineering, Shanghai University of Engineering Science, 201620 Shanghai, P.R. China

^5^ Department of Biosystems and Soft Matter, Institute of Fundamental Technological Research, Polish Academy of Sciences, 02-106 Warsaw, Poland

**Supplementary images and table**


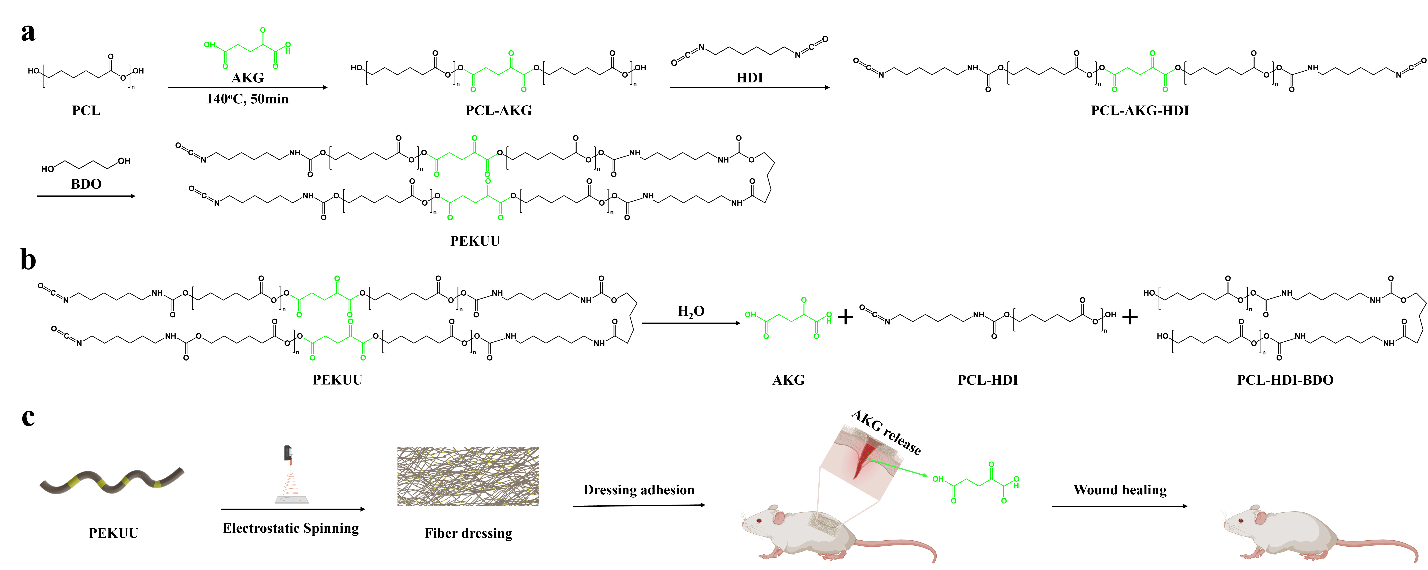


Figure S1. Synthesis and releasing of electrospinning.


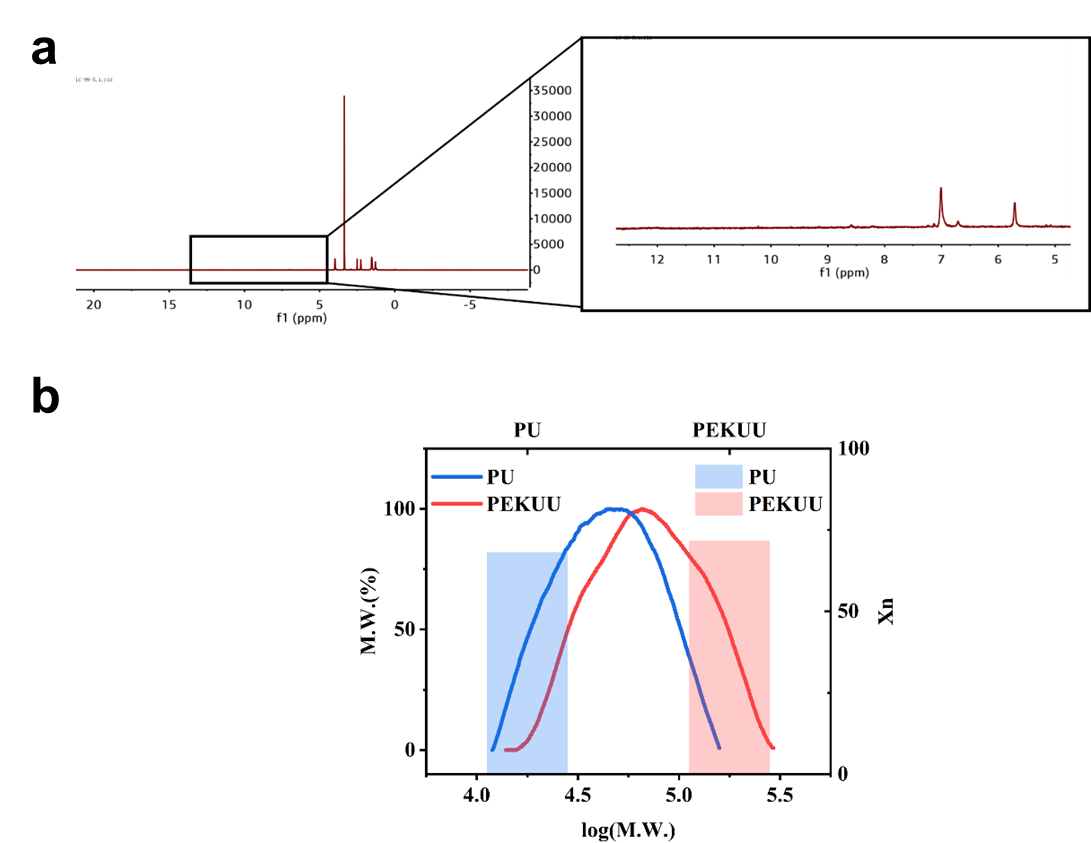


Figure S2. (a) nuclear magnetic resonance hydrogen spectroscopy of PEKUU electrospinning. (b) Gel permeation chromatography profiles of PU and PEKUU fibers.

Figure S3. Cell viability of HUVEC and HaCaT cells after co-incubation with pure medium and electrospinning for 24, 72 hours (n = 4).


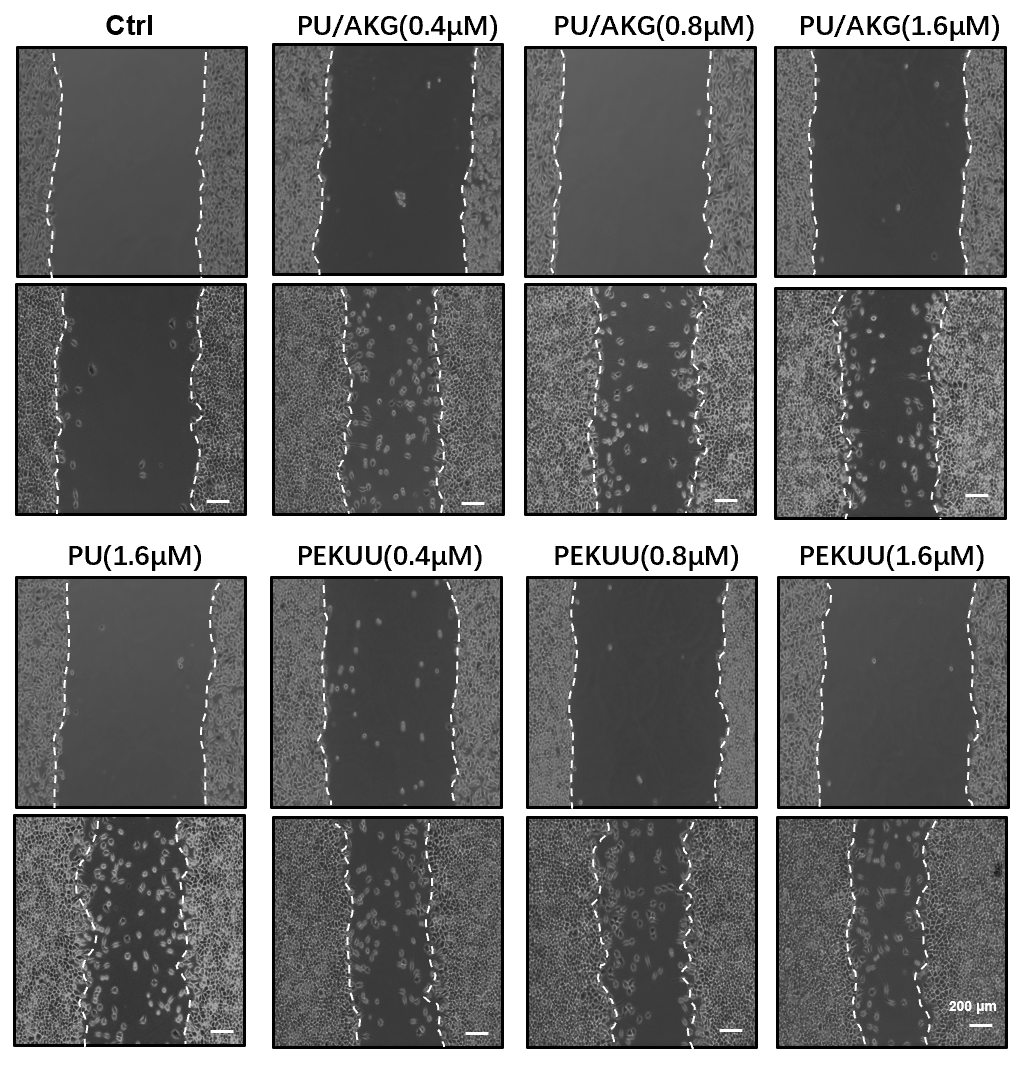

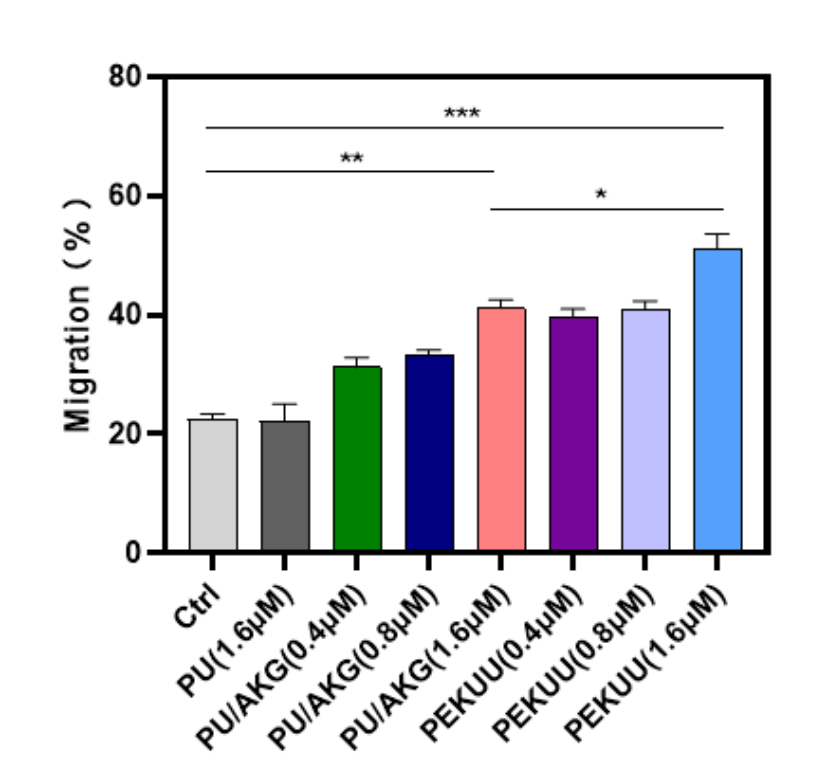


Figure S4. Scratch experiments after treatment with various concentrations of PU, PU/AKG and PEKUU.


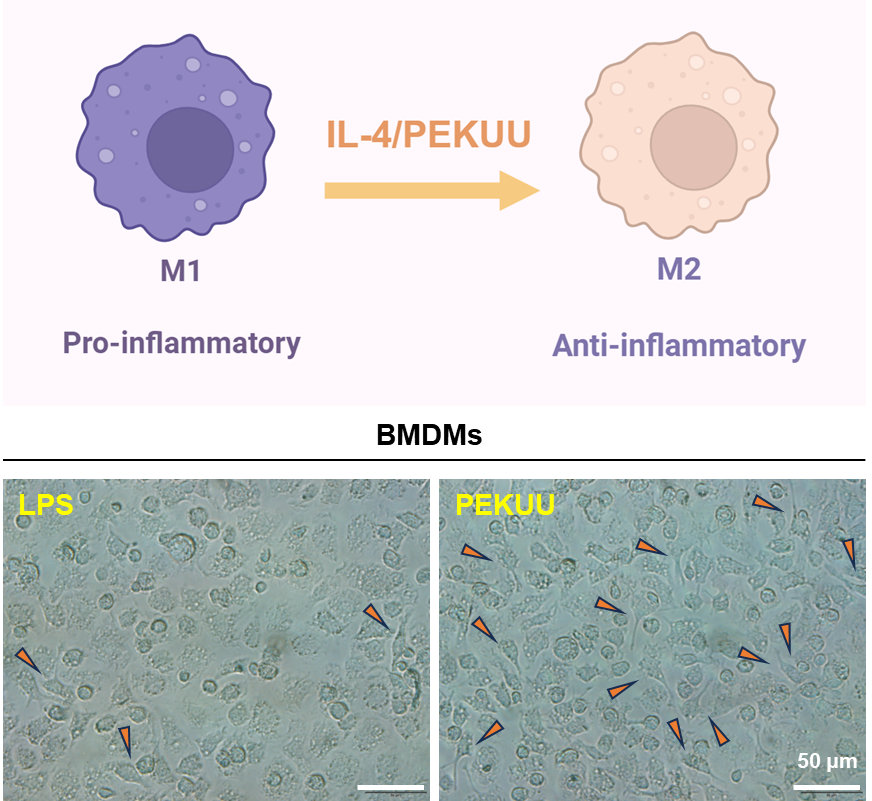


Figure S5. Morphological changes of macrophage after PEKUU electrospinning treatment.


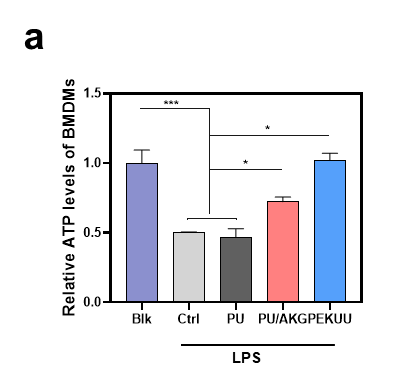


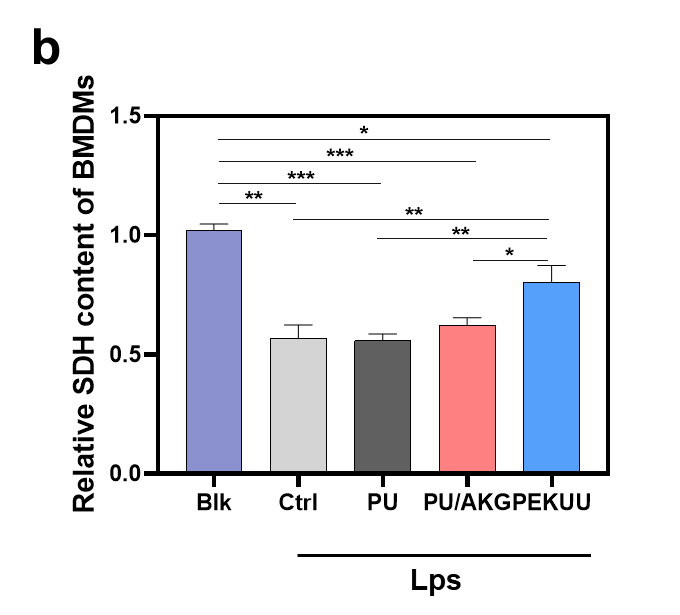


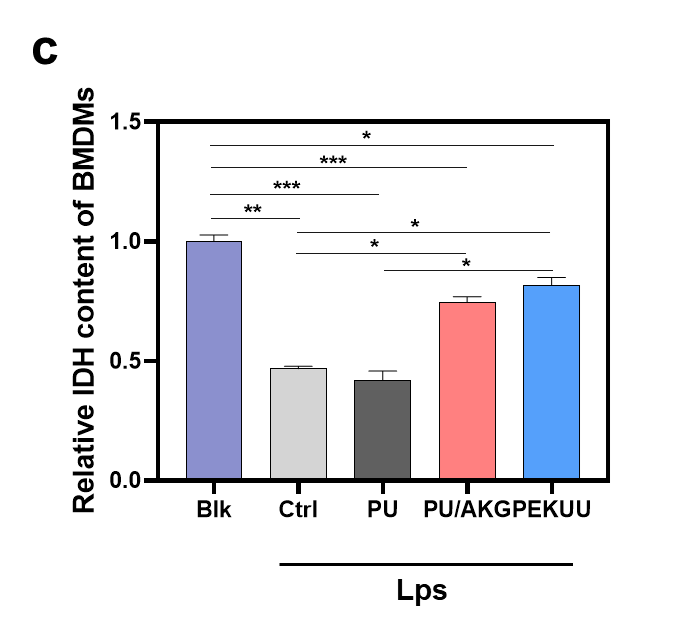


Figure S6. （a)Relative ATP levels of Enzyme linked immunosorbent assay(Elisa)in the blank, Ctrl, PU, PU/AKG , and PEKUU groups (n = 3). (b)Relative SDH contents in the in the negative Ctrl(Blk) ,and PBS,PU,PU/AKG, and PEKUU groups with LPS (n = 3). (c)Relative IDH contents in the in the negative Ctrl (Blk), and PBS, PU, PU/AKG, and PEKUU groups with LPS (n = 3).


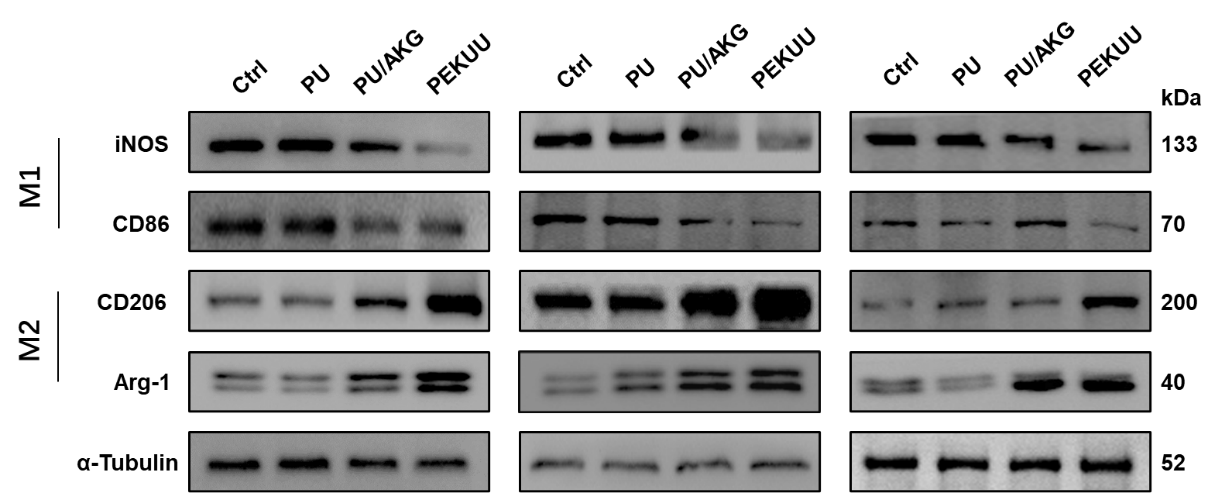


Figure S7. Three Western Blot assay biological repeats of macrophage polarization.


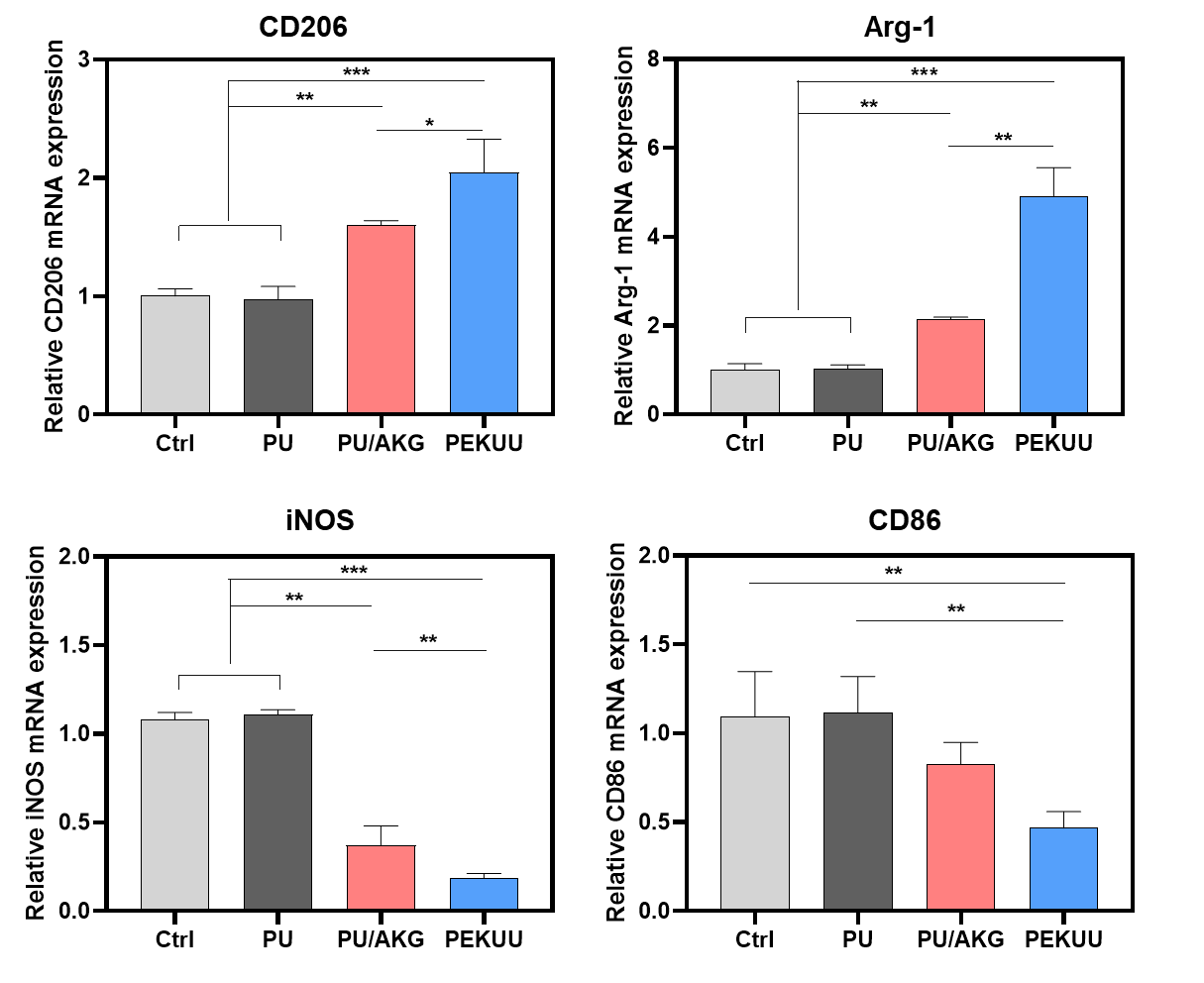


Figure S8. Macrophage polarization of RT-qPCR validation

Relative mRNA expression of Arg-1, iNOS, CD86 and CD206 in the in the Ctrl , PU , PU/AKG , and PEKUU groups (n = 3)


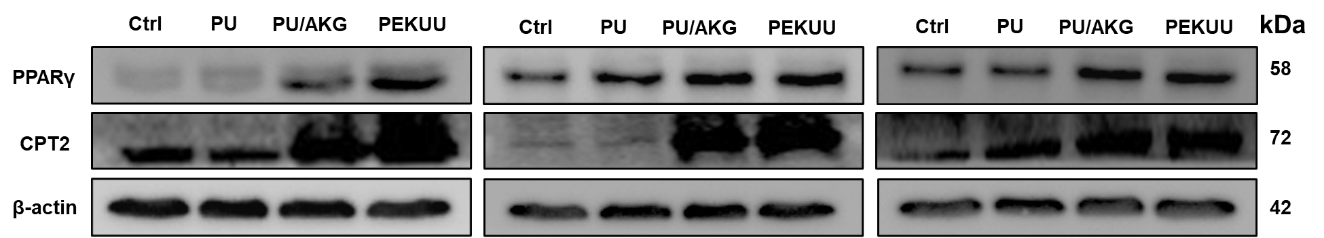


Figure S9. Macrophage fatty acid oxidation (FAO) validation in 3 biological replicates


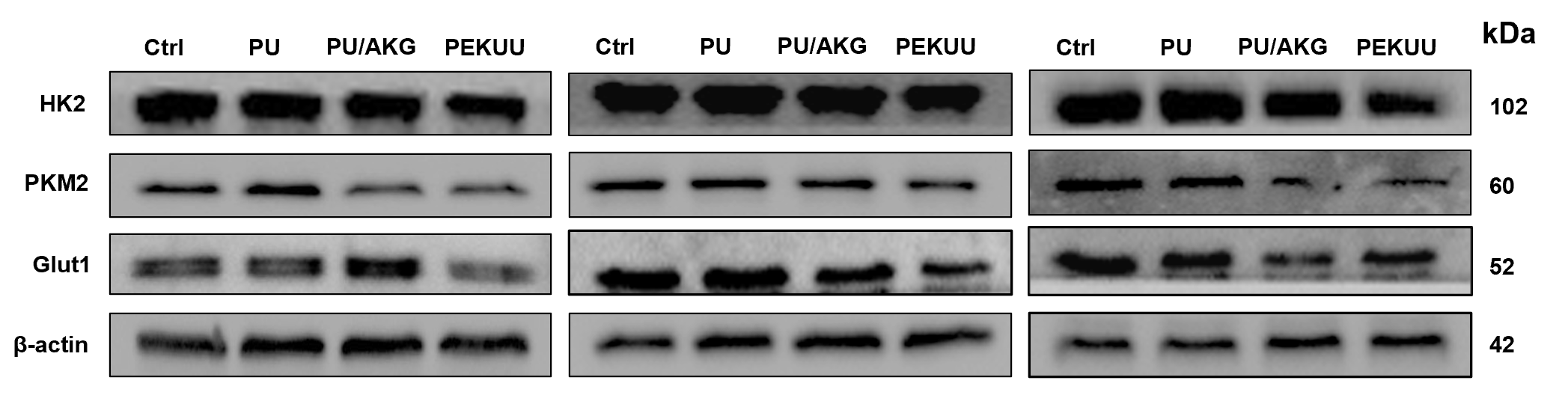
Figure S10. Macrophage glycolysis (Glycolysis) validated in 3 biological replicates


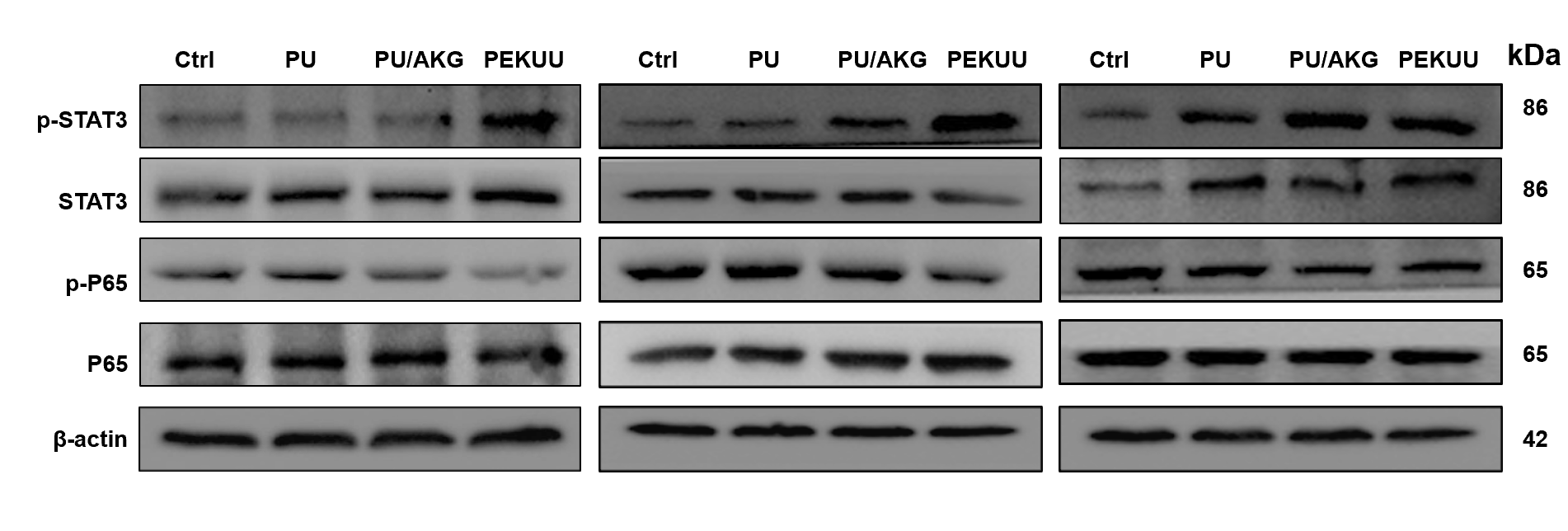
 Figure S11. Macrophage metabolic reprogramming regulates polarization-associated transcription factor (Translation factor) Validation of 3 biological replicates


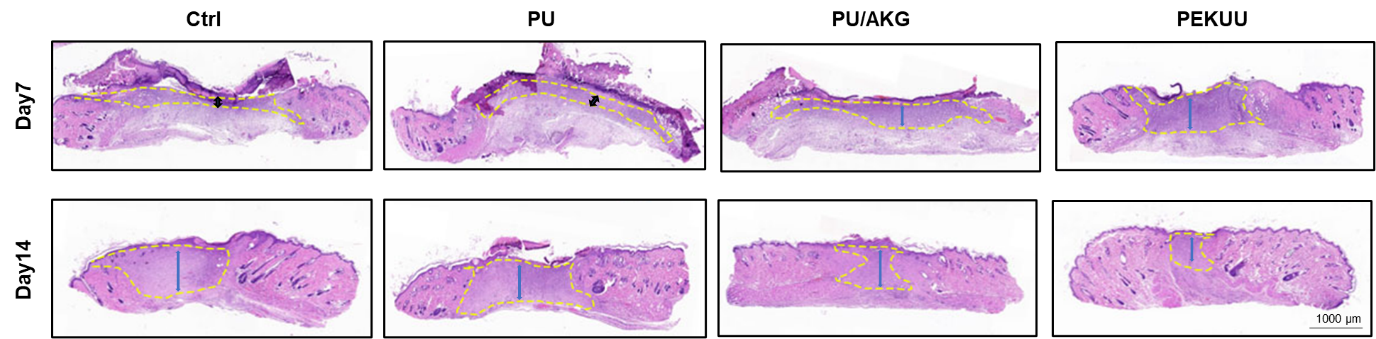


Figure S12. Schematic of granulation tissue thickness

The yellow dotted circle shows the extent of neoplastic granulation tissue, and the blue color indicates the thickness of the granulation tissue.

Since statistical indicators for wound healing should be based on the same sample, this image corresponds to Figure 5h in the main text. We have marked the granulation tissue with differently colored arrows.


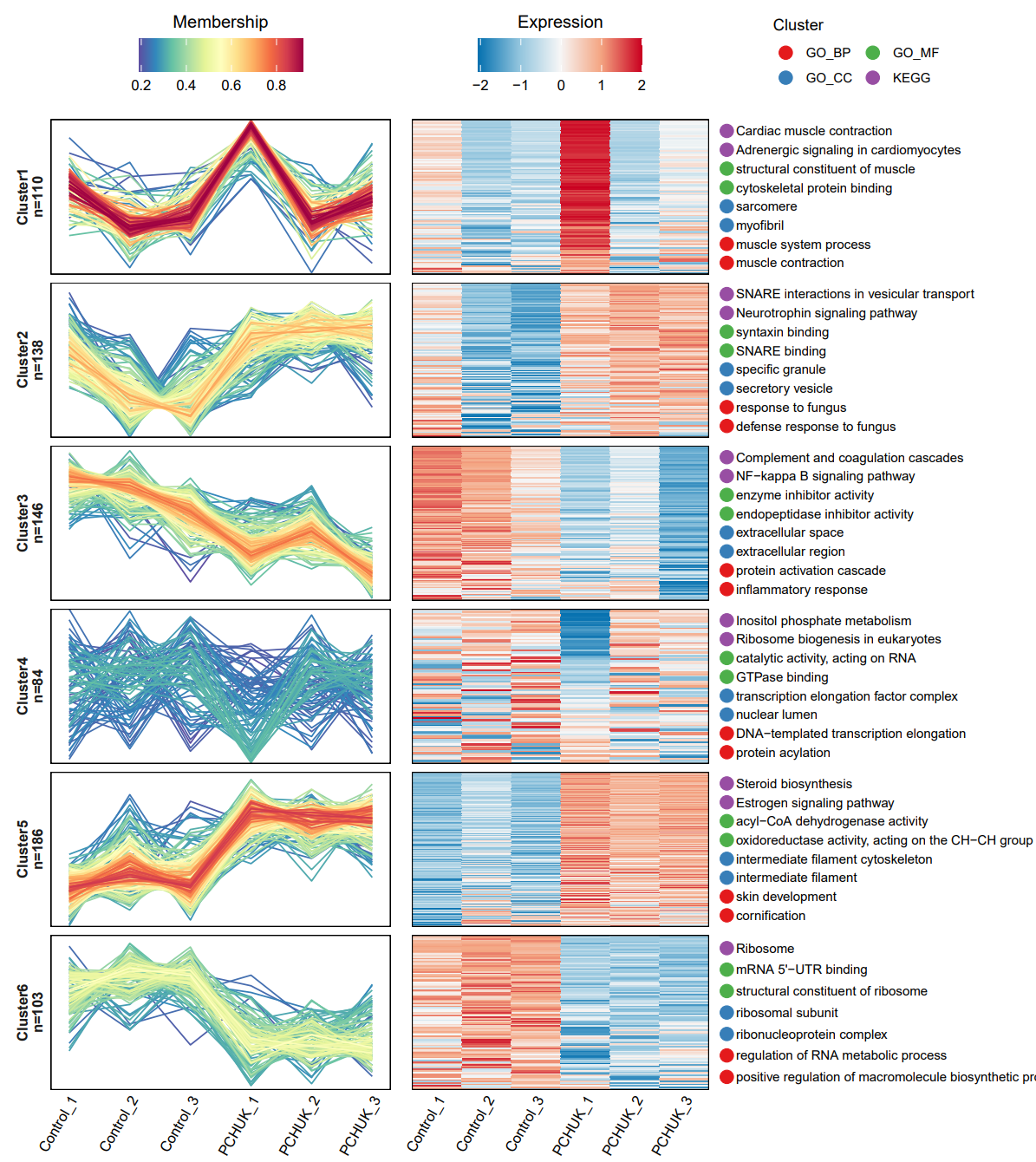


Figure S13. Proteomics cluster analysis of ethnic groups after PEKUU treatment


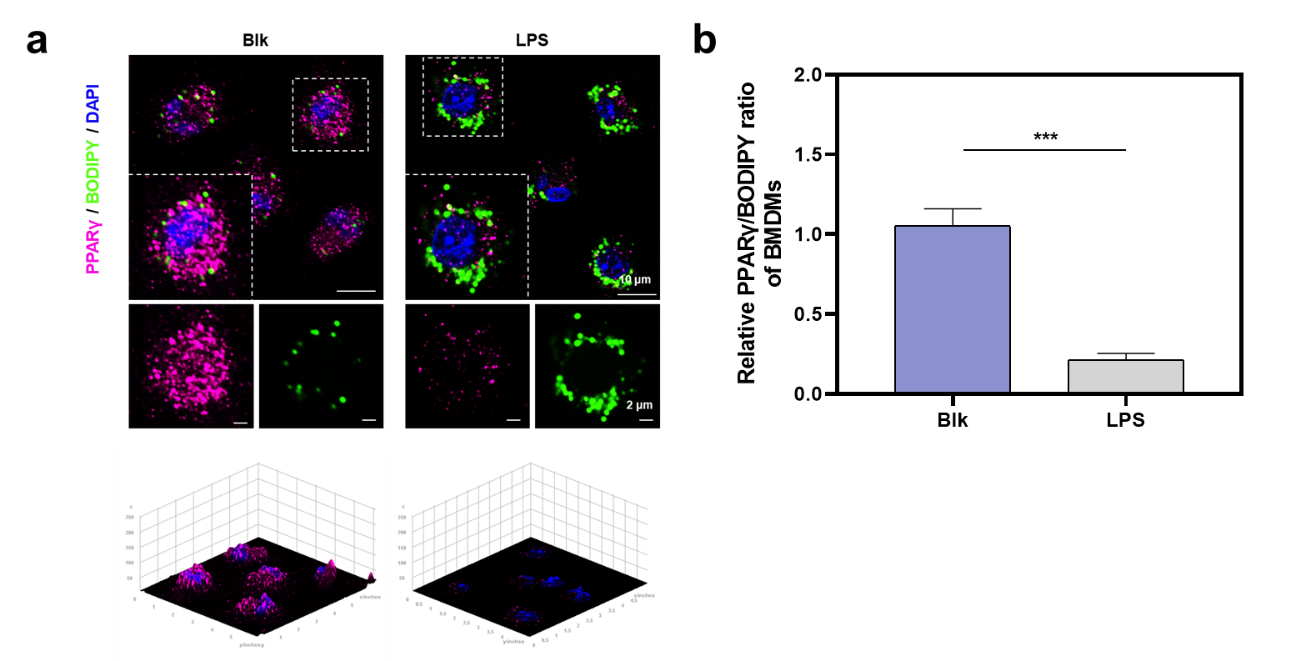
Figure S14. in vitro model of LPS-induced inflammation demonstrates attenuated lipid accumulation by fatty acid oxidation in inflammatory states.

When PPARγ expression was reduced after LPS intervention and BMDM cells showed significant lipid droplet accumulation, from the lower fluorescence projection peak plot, it can be seen that BMDM is reduced by the inflammatory intervention and almost no longer enters the nucleus to play a role in the transcriptional regulation of BMDM, This proves that our use of LPS to induce the in vitro inflammation model is successful and shows that PPARγ is downregulated in the inflammatory state, leading to the accumulation of lipid droplets in BMDM cells.


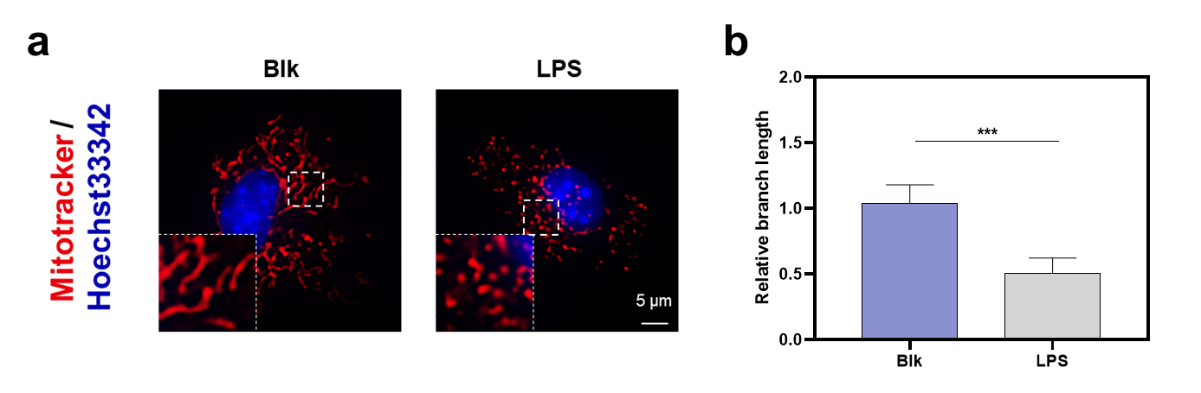


Figure S15. in vitro model of LPS-induced imbalance in mitochondrial dynamics.

Under normal conditions, functional macrophage mitochondria form a regular network of elongated structures accompanied by a small number of fragmented mitochondria. Following LPS stimulation, however, mitochondrial dynamics become imbalanced, with most mitochondria in a fragmented state. This indicates that LPS induces imbalance in mitochondrial dynamics in macrophages.

**Table S1**

Nucleotide primers used for RT-qPCR.

| Abbreviations | Primer sequence (5’-3’) | | |  |  |
| --- | --- | --- | --- | --- | --- |
| CD86 | Forward: AACTTACGGAAGCACCCACG | | |  |  |
|  | Reverse: ATAAGCTTGCGTCTCCACGG | | |  |  |
| CD206 | Forward: GCACTGGGTTGCATTGGTTT | | |  |  |
|  | Reverse: CCTGAGTGGCTTACGTGGTT | | |  |  |
| Arg-1 | Forward: CAGCACTGAGGAAAGCTGGT | | |  |  |
|  | Reverse: ACAGACCGTGGGTTCTTCAC | | |  |  |
| iNOS | Forward: CAGCTGGGCTGTACAAACCTT  Reverse: CATTGGAAGTGAAGCGTTTCG | | |  |  |
| Actb | | | Forward: AAATCGTGCGTGACATCAAAGA  Reverse: GCCATCTCCTGCTCGAAGTC |  | |
